# Dual role of DMXL2 in olfactory information transmission and the first wave of spermatogenesis

**DOI:** 10.1371/journal.pgen.1007909

**Published:** 2019-02-08

**Authors:** Clara Gobé, Maëva Elzaiat, Nicolas Meunier, Marjolaine André, Eli Sellem, Patrice Congar, Luc Jouneau, Aurélie Allais-Bonnet, Ikrame Naciri, Bruno Passet, Eric Pailhoux, Maëlle Pannetier

**Affiliations:** 1 UMR BDR, INRA, ENVA, Université Paris Saclay, Jouy-en-Josas, France; 2 UMR 7592 Institut Jacques Monod, Université Paris Diderot/CNRS, Paris, France; 3 NBO, INRA, Université Paris Saclay, Jouy en Josas, France; 4 Université de Versailles Saint-Quentin en Yvelines, Versailles, France; 5 R&D Department, ALLICE, Paris, France; 6 Epigenetics and Cell Fate, Université Paris Diderot, Sorbonne Paris Cité, UMR 7216 CNRS, Paris, France; 7 UMR-GABI 1313, INRA, AgroParisTech, Université Paris-Saclay, Jouy-en-Josas, France; Monash University, AUSTRALIA

## Abstract

Gonad differentiation is a crucial step conditioning the future fertility of individuals and most of the master genes involved in this process have been investigated in detail. However, transcriptomic analyses of developing gonads from different animal models have revealed that hundreds of genes present sexually dimorphic expression patterns. *DMXL2* was one of these genes and its function in mammalian gonads was unknown. We therefore investigated the phenotypes of total and gonad-specific *Dmxl2* knockout mouse lines. The total loss-of-function of *Dmxl2* was lethal in neonates, with death occurring within 12 hours of birth. *Dmxl2*-knockout neonates were weak and did not feed. They also presented defects of olfactory information transmission and severe hypoglycemia, suggesting that their premature death might be due to global neuronal and/or metabolic deficiencies. *Dmxl2* expression in the gonads increased after birth, during follicle formation in females and spermatogenesis in males. DMXL2 was detected in both the supporting and germinal cells of both sexes. As *Dmxl2* loss-of-function was lethal, only limited investigations of the gonads of *Dmxl2* KO pups were possible. They revealed no major defects at birth. The gonadal function of *Dmxl2* was then assessed by conditional deletions of the gene in gonadal supporting cells, germinal cells, or both. Conditional *Dmxl2* ablation in the gonads did not impair fertility in males or females. By contrast, male mice with *Dmxl2* deletions, either throughout the testes or exclusively in germ cells, presented a subtle testicular phenotype during the first wave of spermatogenesis that was clearly detectable at puberty. Indeed, *Dmxl2* loss-of-function throughout the testes or in germ cells only, led to sperm counts more than 60% lower than normal and defective seminiferous tubule architecture. Transcriptomic and immunohistochemichal analyses on these abnormal testes revealed a deregulation of Sertoli cell phagocytic activity related to germ cell apoptosis augmentation. In conclusion, we show that *Dmxl2* exerts its principal function in the testes at the onset of puberty, although its absence does not compromise male fertility in mice.

## Author summary

*DMXL2* gene dysfunction underlies various human diseases, including breast cancer, non-syndromic hearing loss, and polyendocrine-polyneuropathy syndrome, demonstrating the large range of potential actions of DMXL2. We show here that *Dmxl2* expression is crucial for survival in mice, as neonates die within hours of birth when this gene is inactivated. The transmission of olfactory information is affected, leading to an absence of suckling and impaired feeding. Severe hypoglycemia is also observed in male neonates. We observed *Dmxl2* expression in several organs, including the brain, heart and adrenal glands, potentially corresponding to some of the phenotypes observed in *Dmxl2*-deficient pups. We also described *Dmxl2* expression in the reproductive tracts and gonads and showed that *Dmxl2* inactivation specifically in the testes has a significant effect on the initial waves of spermatogenesis, resulting in very low levels of sperm production at puberty. Our results suggest that DMXL2 deficits should be considered in men with impaired fertility, as pathogenic variants of this gene may be associated with male infertility in humans.

## Introduction

The *DMX-Like 2* (*DMXL2*) gene encodes a protein with multiple WD40 domains, which mediate protein-protein and protein-DNA interactions [1], [2], [3]. These WD40 domains correspond to a series of 44- to 60-amino acid sequence repeats with a characteristic sequence terminating in a tryptophan–aspartic acid (WD) dipeptide [4]. WD40 proteins function as platforms, recruiting multiple partners for a wide range of cellular functions, such as signal transduction, vesicular trafficking, cell-cycle control, chromatin dynamics and the regulation of transcription [2], [3]. Little is known about DMXL2 and its various functions in different species. This protein was first described as rabconnectin-3α (or Rbcn-3α) in the rat brain, where it forms the stoichiometric rabconnectin-3 complex with WDR7 (rabconnectin-3β or Rbcn-3β) [5]. In the brain, DMXL2 has been implicated in the regulation of neurotransmitter exocytosis via its interactions with the small G-proteins Rab3-GEP and Rab3-GAP [1], [6]. The second main function attributed to DMXL2 is modulating vacuolar-ATPase proton pump (V-ATPase) assembly and activity, through interactions with several of its subunits [7], [8]. This function is highly conserved and has been described in numerous species, including yeast [9], [10], *Drosophila* [7], zebrafish [11], mice [12] and humans [13], [14]. V-ATPases acidify intracellular compartments (e.g. endosomes, lysosomes, and secretory vesicles) and they play a well-established role in protein sorting, trafficking and turnover in a wide range of signaling pathways, including the Notch pathway [15], [16]. DMXL2 has been shown to be involved in the Notch signaling pathway [7], [13], [14], [17] during diverse morphogenetic processes, such as the formation of ovarian follicles in the female gonads in *Drosophila* [7]. A role for this protein in reproductive function has also been suggested in humans, in which DMXL2 has been implicated in a complex syndrome of congenital hypogonadotropic hypogonadism (CHH) associated with polyneuropathy and glucose metabolism disorders [18]. Three brothers in the family concerned were affected and presented incomplete puberty with a low testicular volume. Their testosterone and gonadotropin (LH, FSH) levels were low, due to a dysfunction of gonadotropin-releasing hormone (GnRH) neurons. In mice, decreasing the level of *Dmxl2* expression in neurons (*Dmxl2*^wt/loxP^ *; nes-Cre*) resulted in 30% fewer GnRH neurons [18] and an impairment of their activation and maturation [19]. The fertility of these mice also seemed to be impaired, consistent with a hypothalamic dysfunction.

Several clues to the functions of DMXL2 have emerged from studies of these models, but *Dmxl2* knockout studies have never been reported. Furthermore, DMXL2 has never been directly implicated in the functions of reproductive organs in mammals. We show here that a complete loss-of-function of *Dmxl2* results in neonatal lethality within a few hours of birth. *Dmxl2*-knockout pups present defects of olfactory information transmission associated with an absence of feeding. A glucose metabolism disorder was also detected, with male *Dmxl2* KO pups displaying severe hypoglycemia. A description of the expression profile of *Dmxl2*/DMXL2 revealed a possible role in ovary and testis function. We therefore studied the specific effects of *Dmxl2* knockout in the germ cells and supporting cells of the gonads of both sexes. Long-term fertility appeared to be unaffected in both sexes, but young male knockout mice produced less sperm than wild-type controls at the beginning of their reproductive life.

## Results

### *Dmxl2* knockout is lethal in neonates

**Fig 1:**
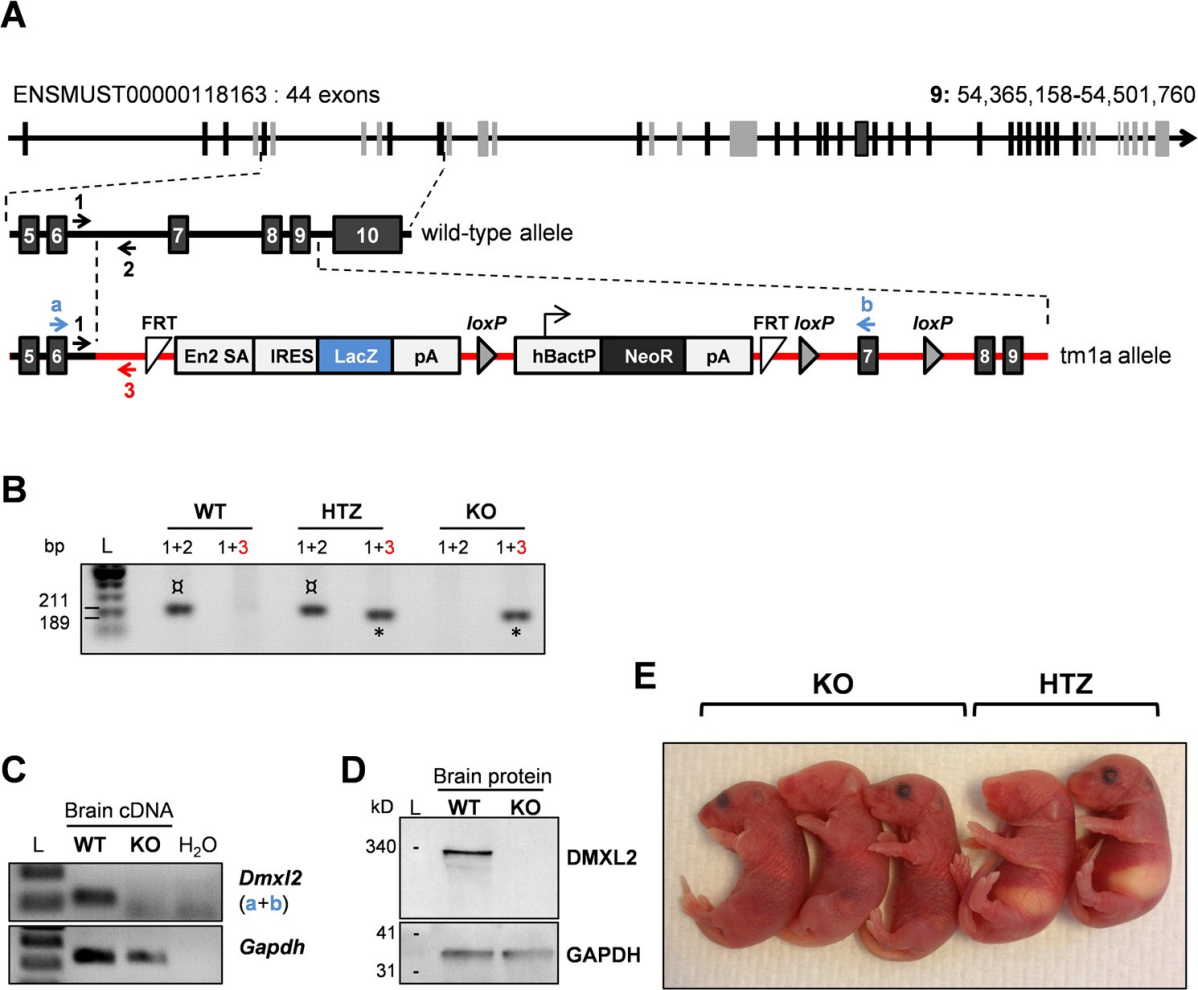
Generation of *Dmxl2* KO mice. **(A)** Genetic construct. *Dmxl2* is located on chromosome 15 in mice, and encodes 9 isoforms, 4 of which are reported to be protein-coding in Ensembl (ENSMUSG00000041268). The longest form is represented here; it has 44 exons containing 13 WD40 domains according to SMART (exons encoding WD40-domains are shown in gray). Heterozygous mice (*Dmxl2*^*wt/tm1a*^) were obtained from the International Mouse Phenotype Consortium http://www.mousephenotype.org. The mutation corresponded to the insertion of the targeting vector between *Dmxl2* exons 6 and 10 (red line). The targeting vector contained a splice acceptor site and an IRES in *Dmxl2* intron 6 upstream from the *LacZ* reporter gene, and a polyadenylation signal downstream from *LacZ*. Consequently, *Dmxl2* transcripts truncated after exon 6 should be produced and *LacZ* should be expressed under the control of the endogenous *Dmxl2* promoter. *FRT*: flippase recognition target; *En2* SA: splice acceptor of mouse *Engrailed2* exon 2; *IRES*: internal ribosome entry site; *LacZ*: gene encoding β-galactosidase; *pA*: polyadenylation signal; *loxP*: sequence targeted by Cre recombinase; *hBactP*: human β-actin gene promotor; *Neo*: neomycin resistance gene. **(B)** PCR genotyping on mouse DNA yielded the following amplicon sizes: 211 bp for the wild type; 211 and 189 bp for heterozygous mice; and 189 bp for KO mice. The two amplicons are indicated by different symbols as their sizes on the gel are very similar (¤ and * for 211 bp and 189 bp, respectively). **(C)** Potential residual expression of the *Dmxl2* wild-type sequence in tissues with a knockout (KO) of the gene was assessed by PCR. The primers “a” and “b” used for this purpose are represented by blue arrows on the genetic construct diagram. No amplification was obtained with brain cDNA from KO mice. **(D)** Western blotting detected none of the four protein isoforms (341, 338, 249 and 146 kDa), confirming the efficacy of *Dmxl2* invalidation in KO animals and antibody specificity. (**E**) Gross morphology of newborn knockout (KO) and heterozygous (HTZ) mice; note the empty stomach of KO animals.

Heterozygous *Dmxl2*^*tm1a(EUCOMM)Wtsi*^ mice were purchased from the IMPC. These mice carry a recombinant allele (tm1a) containing an *IRES*:*LacZ* trapping cassette and a promoter-driven neo cassette in intron 6 flanked by two FRT sites. Two loxP sites are also present, one at either end of exon 7 of *Dmxl2*. Transcription of the tm1a allele generates a truncated/abnormal *Dmxl2* mRNA and leads to *LacZ* gene expression (Fig 1A) [20], [21], [22]. Crosses of *Dmxl2*^*tm1a(EUCOMM)Wtsi*^ mice generated *Dmxl2*^*wt/wt*^ *(*wild-type, WT), *Dmxl2*^*wt/tm1a*^ (heterozygous, HTZ) and *Dmxl2*^*tm1a/tm1a*^ (knockout, KO) offspring in Mendelian proportions. The pups were genotyped with a combination of primers provided by the IMPC (Fig 1B and S1 Table). Neither a functional *Dmxl2* transcript (Fig 1C), nor DMXL2 protein (Fig 1D) was produced in the organs of newborn KO mice such as the brain, demonstrating the efficiency of the knockout. KO newborns died within 12 hours of birth. KO pups were of normal size and skin color at birth (Fig 1E), but they looked weaker than control littermates, with poor motility, and their stomachs contained no milk, suggesting that they did not feed.

### *Dmxl2* is expressed in various tissues during development

**Fig 2:**
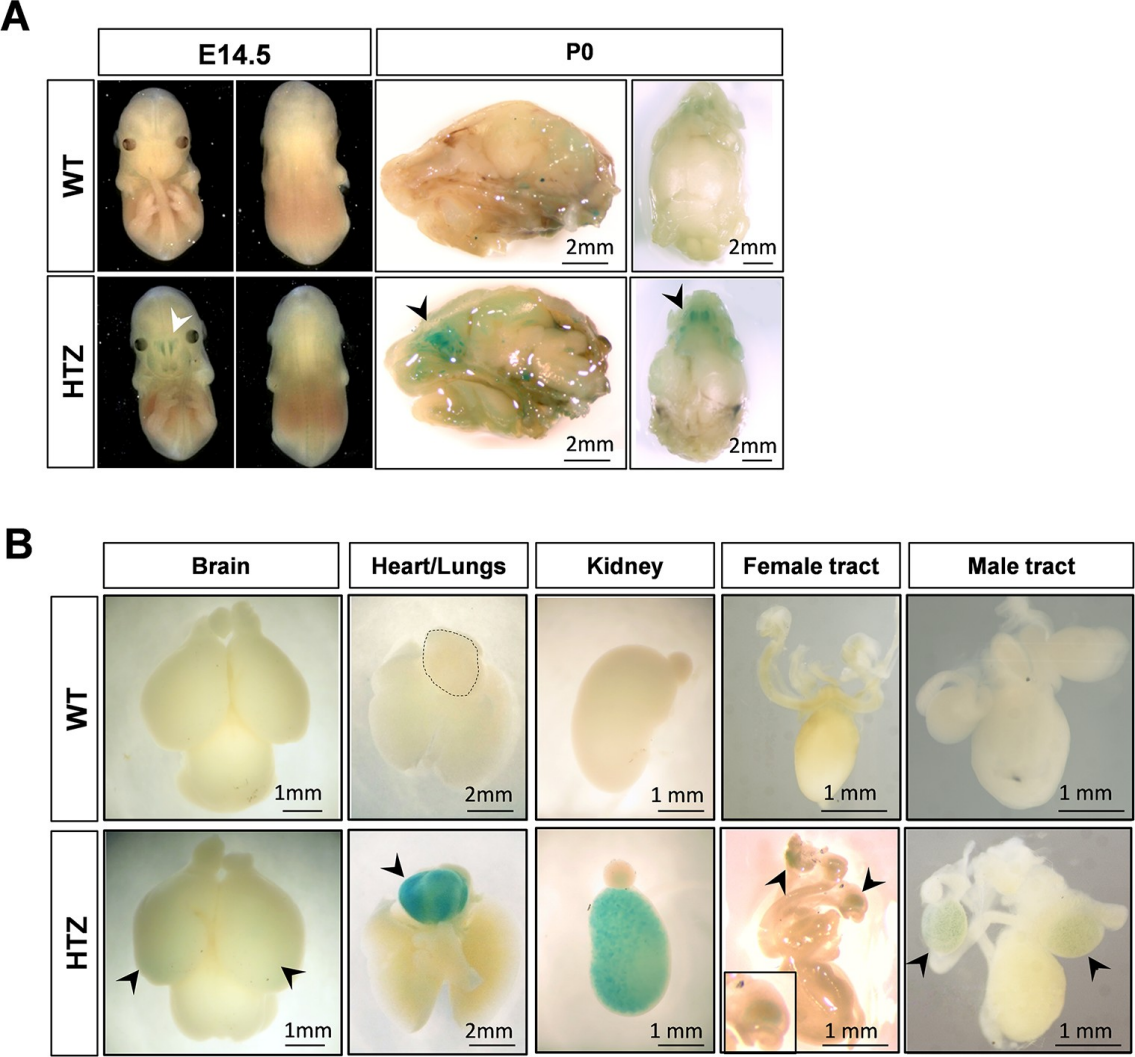
*Dmxl2* expression during fetal development and at birth, as assessed by whole-mount X-gal staining. **(A)** X-gal staining of E14.5 embryos and P0 heads. Staining revealed a restriction of *Dmxl2* expression to the olfactory mucosa at E14.5 (arrowhead). This gene was still strongly expressed in this epithelium at birth. **(B)** X-gal staining of the brain, heart and lungs, kidneys and adrenal glands, and female and male genital tracts at P0. Diffuse staining was observed in the cerebral cortex of both hemispheres (arrowheads). Specific staining was also detected in the heart, kidneys, ovaries and testes (arrowheads).

**Fig 3:**
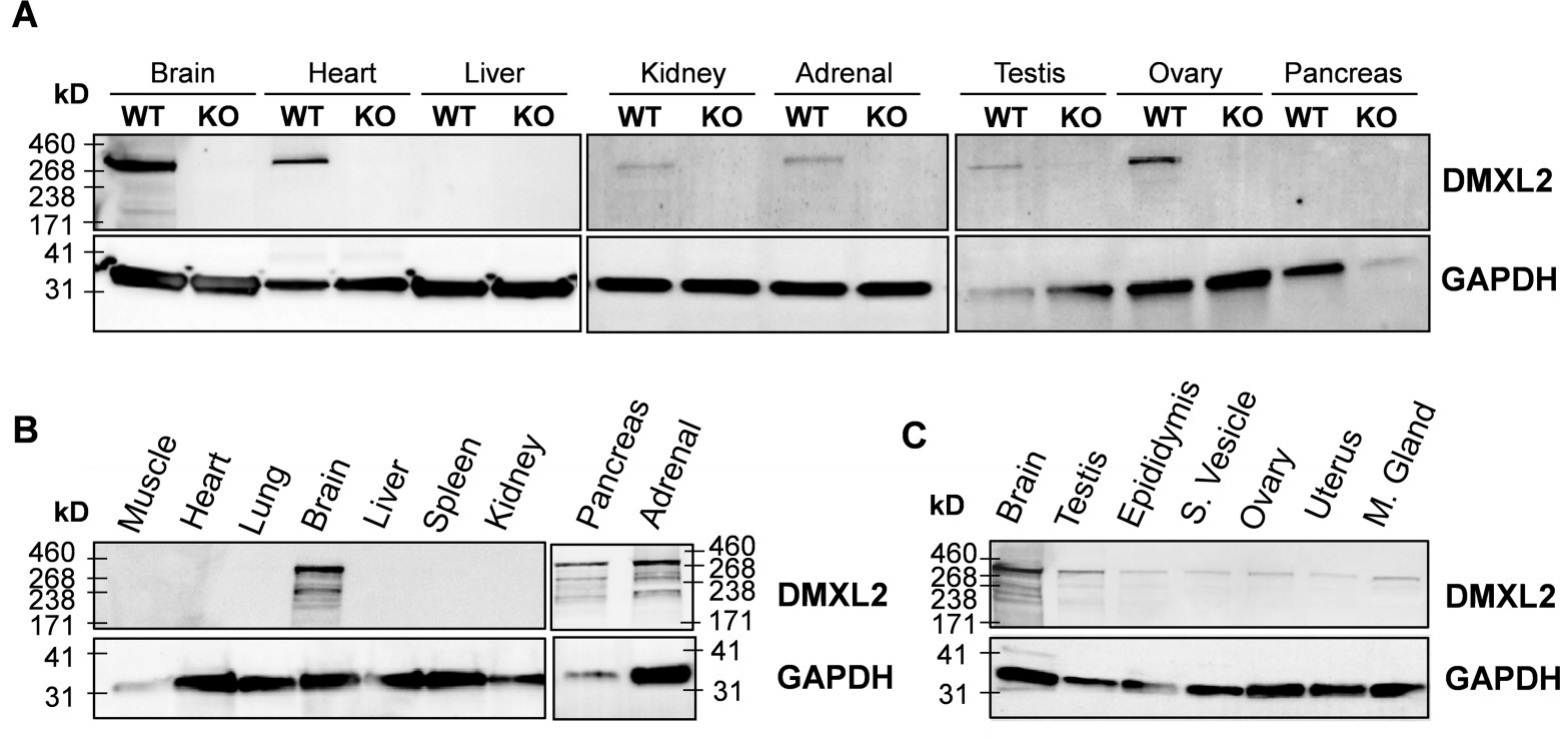
DMXL2 protein in newborn and adult tissues. **(A)** DMXL2 levels at P0 in WT and KO tissues. Brain, heart, kidneys, adrenal glands, testes and ovaries displayed specific DMXL2 expression at birth, whereas western blotting detected no protein in the liver or pancreas. **(B)** In adults, DMXL2 was strongly detected in the brain and was also found in the pancreas and adrenal glands on western blots. By contrast, DMXL2 was not detected in adult skeletal muscles, heart, lungs, liver, spleen or kidneys. **(C)** DMXL2 was also detected in adult testes, and gave a weaker signal in the epididymides, seminal vesicles, ovaries, uterus horns and mammary glands (M. gland).

We investigated the tissue expression profile of *Dmxl2*, by assessing *LacZ* reporter gene expression in mutants (whole-mount X-gal staining at E14.5 and P0) and by western blotting (at P0 and in adults). At E14.5, specific X-gal staining was restricted to the olfactory mucosa (Fig 2A). At birth (P0), prominent staining persisted in the olfactory mucosa (arrowhead) (Fig 2A) but specific X-gal staining was observed in other tissues (Fig 2B). Indeed, detailed dissection and fixation of the brain revealed faint, diffuse and scattered staining of both hemispheres of the cerebral cortex, confirming the expression of *Dmxl2* in this organ [18]. The heart and kidneys were also strongly stained, and the gonads of both sexes displayed punctate staining, with enrichment in the cortical region of the ovaries, and in the seminiferous cords of the testes (Fig 2B). We also tested various organs, including the liver, pancreas, digestive tract, adrenal glands, and skeletal muscles. These tissues displayed no staining or only nonspecific staining, like the WT control tissues. Western blots comparing WT and KO organs confirmed that DMXL2 was expressed specifically in the brain, heart, kidney and gonads of both sexes at P0, whereas no expression was observed in the liver and pancreas (Fig 3A). In addition, DMXL2 expression was detected specifically in the adrenal glands at P0, but these glands displayed no X-gal staining.

In adults, the DMXL2 protein was detected in large amounts in the brain, as previously reported in rats [1], but also in the pancreas and adrenal glands (Fig 3B). In addition to the larger described isoforms that predominate (341 kD and/or 338 kD), two or three smaller isoforms (180 to 250 kD) were detected, depending on the tissue (and the level of DMXL2 expression in the organ concerned). Weaker, but clearly detectable expression was also observed in the testes, epididymides, seminal vesicles, ovaries, uterus horns and mammary glands (Fig 3C). Intriguingly, X-gal staining and western blotting demonstrated the presence of DMXL2 in the heart and kidneys at P0 (Figs 2C and 3A), but this protein was no longer detectable in these organs in adult animals (Fig 3B). We performed morphometric analyses of E18.5 *Dmxl2* KO embryos to investigate possible developmental defects of the heart and kidneys that might explain neonatal death. However, these studies revealed no cardiac or renal malformations or any other organ defects capable of accounting for the premature death of these mice (observations made by Prof. Manuel Mark, IGBMC, Illkirch).

*Dmxl2/DMXL2* has been implicated in glucose metabolism [18]. We therefore assessed the blood glucose and plasma insulin concentrations of newborn pups of the different genotypes (S1 Fig). These concentrations were normal for female KO pups, but male KO pups were hypoglycemic (pValue ≤ 0.001) relative to their WT and HTZ littermates, despite normal insulinemia. As hypoglycemia affected the male pups only and neonatal lethality displayed no sex bias, it appears unlikely that this feature is responsible for the lethality of *Dmxl2* knockout.

Strong *Dmxl2* expression was detected in the olfactory mucosa as early as E14.5, and the newborn KO pups did not feed. We therefore investigated the possible effects of the loss of function of this gene on the olfactory system.

### DMXL2 is involved in transmitting information from the olfactory mucosa to the olfactory bulb

**Fig 4:**
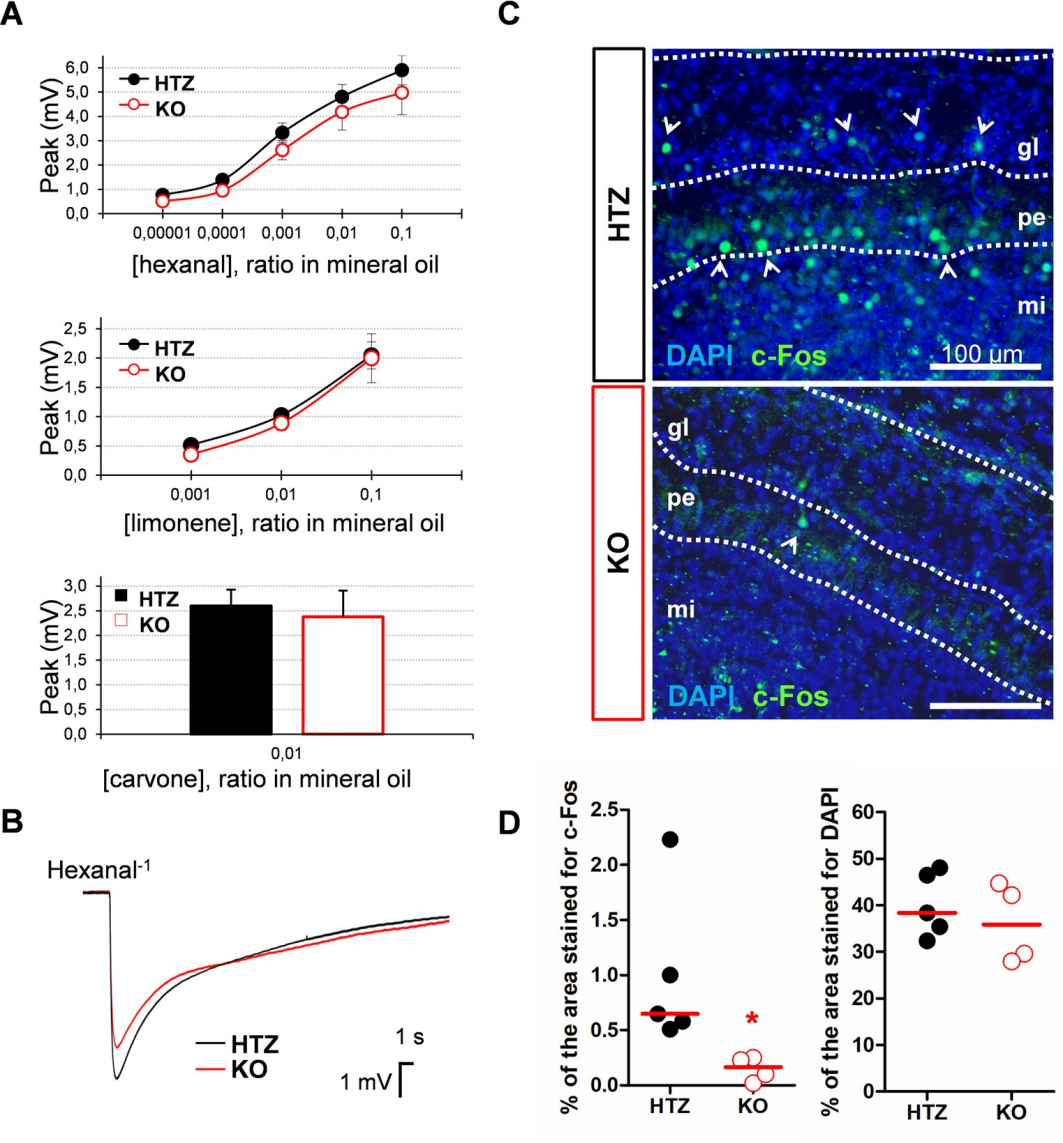
Changes in synaptic transmission but not odorant detection in the olfactory system of newborn *Dmxl2-*knockout mice. **(A)** EOG peak amplitudes in response to various odorants were recorded at P0 in newborn HTZ (black dots) and KO (red dots) pups. No significant differences were found, demonstrating the ability of the olfactory mucosa of newborn *Dmxl2* KO mice to detect odorants (Fisher-Pitman permutation test). **(B)** Cumulative traces of HTZ (black) and KO (red) EOG responses to hexanal diluted 1:10 in mineral oil. **(C)** Immunodetection of c-Fos in HTZ and KO olfactory bulbs after odorant stimulation. C-Fos-positive neurons (white arrowheads) were more abundant in HTZ than in KO olfactory bulbs (gl: glomerular cell layer; pe: plexiform external cell layer; mi: mitral cell layer). **(D)** Quantification of c-Fos activation and neuron density (DAPI signal) in the olfactory bulb. Specific c-Fos (left panel) and DAPI (right panel) signals were defined on the basis of the size and shape of the area stained. These signals are reported relative to the area of the plexiform external region and glomerular cell layers. Median values were plotted, and statistical analyses were performed with Fisher-Pitman two-sample exact permutation tests (pValue<0.05). Significant differences are indicated by an asterisk (pValue = 0.023).

The olfactory system is known to regulate feeding behavior [23]. We therefore performed electro-olfactography (EOG) to investigate the functionality of the olfactory mucosa in KO pups. EOG signals result from the activation of the olfactory transduction cascade in a population of neurons located close to the recording electrode. This transduction cascade occurs in the neuronal cilia in contact with their environment. We stimulated the olfactory mucosa with various odorants, at several concentrations (Fig 4A). The maximum amplitude of the response to odorants was measured and did not differ significantly between KO and HTZ (control) pups (Fig 4A and 4B). The olfactory mucosa was, therefore, functional, and the peripheral olfactory sensory neuron cilia of KO pups were as capable of odorant detection as those of control newborn mice.

DMXL2 is associated with synaptic vesicles in rat brain, potentially regulating their exocytosis and signal transmission [1], [11]. We therefore investigated whether the olfactory information generated in the olfactory mucosa was efficiently transmitted to the neurons of the olfactory bulb. Neuronal activation in the olfactory bulb after odorant stimulation was assessed by c-Fos immunodetection in HTZ and KO pups (Fig 4C and 4D). We observed significantly fewer c-Fos-positive neurons in the glomerular and external plexiform layers of KO olfactory bulbs than in those of HTZ bulbs, whereas neuron density was similar (Fig 4D). The synaptic transmission of the olfactory signal from the olfactory mucosa to the olfactory bulb was, therefore, significantly altered in the absence of *Dmxl2*.

### *Dmxl2* is expressed in both ovaries and testes during fetal and postnatal development

**Fig 5:**
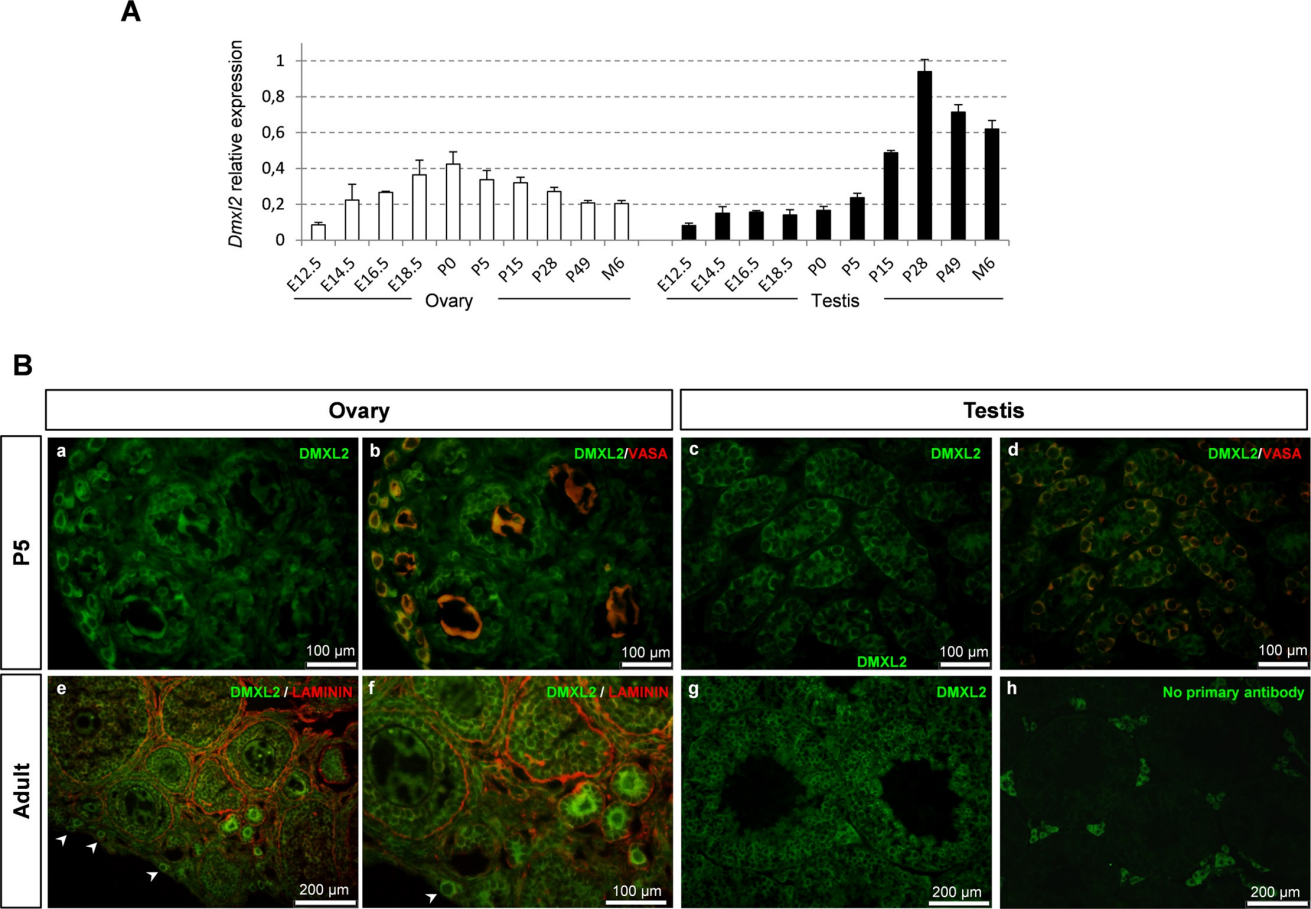
Characterization of *Dmxl2*/DMXL2 expression during ovarian and testicular development. **(A)** *Dmxl2* mRNA levels were assessed by RT-qPCR during ovarian (white bars) and testicular (black bars) development. Values are expressed as a proportion of the maximum value observed (the maximum value being set at 1). Means ± SD were plotted. **(B)** Localization of DMXL2 protein in P5 and adult gonads. (a-d) At P5, co-immunodetection experiments were performed for DMXL2 (cytoplasmic green staining) and VASA (cytoplasmic, red staining). (b, d) VASA staining was observed specifically in germ cells of all stages in both male and female gonads. (a,b) In P5 ovaries, DMXL2 was highly abundant in the oocytes of primordial to secondary follicles, and in the granulosa cells. (c,d) In P5 testes, DMXL2 was detected in the seminiferous cords: in the germ cells and the Sertoli cells. (e, f) In adult ovaries, co-immunodetection experiments were performed for DMXL2 (green) and LAMININ (red). The DMXL2 signal remained strong in the oocytes of the primordial and primary follicles. Faint staining was observed in granulosa cells, at least until the preantral stage. (g, h) In adult testes, DMXL2 was detected mostly in the germ cells. Nonspecific staining of the interstitial tissue was observed, as demonstrated by the experiment in which the primary antibody was omitted (h). Arrowheads indicate DMXL2-positive oocytes of primordial follicles.

LacZ staining and western-blotting experiments showed that *Dmxl2*/DMXL2 was expressed in the gonads of both sexes (Figs 2 and 3). Studies of its transcription during gonad differentiation revealed a dynamic profile, with increases at sex-specific stages (Fig 5A). Male and female gonads displayed similar levels of *Dmxl2* transcripts at early stages of differentiation (E12.5), but *Dmxl2* levels increased in the ovary a couple of days before birth (between E16.5 and E18.5: pValue = 0.019), reaching maximum values on P0, before decreasing slightly. *Dmxl2* transcript levels remained low in the adult ovary. The first few days after birth correspond to the breakdown of germ cell nests and the formation of the first follicles in mice [24].

In the testes, *Dmxl2* transcript levels remained constant during fetal and early postnatal development, but increased markedly with the initiation of spermatogenesis after P5 (Fig 5A). Indeed, *Dmxl2* transcript levels had already increased by P15, when early-stage pachytene spermatocytes are observed [25], and they peaked at P28, when the first elongating spermatids are detected [25]. *Dmxl2* transcript levels remained constant thereafter in the adult testes.

Having demonstrated the specificity of the DMXL2 antibody (S2 Fig), we used immunodetection methods to detect the DMXL2 protein at various stages of gonadal differentiation (P5 and P28). A strong signal was detected in the germ cell cytoplasm in both male and female gonads (Fig 5B). A faint signal was also observed in the supporting cells of both sexes (i.e. granulosa and Sertoli cells).

### Despite normal morphology at birth, *Dmxl2* KO gonads display gene dysregulation

As KO pups died shortly after birth, gonad phenotype could be analyzed only on P0. The morphological appearance of the gonads of female and male KO mice was assessed by classical histology methods (S3 Fig) and by the use of several markers of germ cells and supporting cells. Gonad size, organization and general appearance were similar in KO and control gonads. In particular, KO ovaries had numerous germ-cell nests at the cortex and a few primordial follicles were starting to form, as in P0 control ovary (S4 Fig).

Transcriptomic analyses were performed at P0. Microarray analyses comparing KO and WT ovaries and testes highlighted only a few genes differentially expressed between KO and WT gonads: 51 genes for KO ovaries (S2 Table), and 12 for KO testes (S3 Table) (adjusted pValue <0.1). Four of these genes were differentially expressed in the KO gonads of both sexes, as confirmed by RT-qPCR analyses for *Aph1b* and *Fez1* (S5 Fig).

Despite the small number of differentially expressed genes in KO ovaries, two gene clusters with significant enrichment scores were identified (DAVID analysis tool; enrichment score ≥1.3) [26]. One of these gene clusters related to stress responses (enrichment score = 1.42), whereas the other concerned WD40 proteins (enrichment score = 1.68). Indeed, in *Dmxl2* KO ovaries, transcript levels for three other WD40 protein-encoding genes were affected according to the microarray data, which were confirmed by RT-qPCR for *Coro2b*, and *Fbxw8* (S5 Fig). In addition, *Coro2b* transcript levels were found to be upregulated in KO testes. This upregulation was not detected in global analyses.

In conclusion, *Dmxl2* loss-of-function at P0 induced the dysregulation of a larger number of genes in female than in male gonads. Nevertheless, the morphology of KO ovaries was unaffected, with primordial follicle formation occurring as in the control. We evaluated the effect of *Dmxl2* loss-of-function at later stages, including spermatogenesis in the male gonad in particular, by generating mice with conditional knockouts of *Dmxl2* in germ cells and/or in supporting cells of both sexes.

### *Dmxl2* loss of function in gonads does not affect long-term fertility in mice

We obtained conditional knockouts of *Dmxl2* by first generating *Dmxl2*^*loxP/loxP*^ mice (exon 7 floxed) by crossing *Dmxl2*^*wt/tm1a*^ mice with *FlpO* (FLP) recombinase-expressing mice (*Rosa26-FlpO*) (Fig 1A) [27]. We then used several lines of *Cre*-expressing mice: a *Vasa-Cre* line, to generate a conditional *Dmxl2* KO in germ cells (*Dmxl2*^loxP/-^; *Vasa-Cre*: germ cell cKO) [28], *Amh-Cre* [29], to produce a conditional *Dmxl2* KO in Sertoli cells (*Dmxl2*^*loxP/loxP*^; *Amh-Cre*: Sertoli cell cKO) and *Amhr2-Cre* [30] for conditional *Dmxl2* KO in granulosa cells (*Dmxl2*^*loxP/loxP*^; *Amhr2*^*wt/Cre*:^: granulosa cell cKO). Double conditional knockouts (dcKO) were also generated, resulting in Sertoli and germ cell-specific *Dmxl2* dcKO for males (*Dmxl2*^loxP/-^; *Vasa-Cre*; *Amh-Cre*) or granulosa and germ cell-specific *Dmxl2* dcKO for females (*Dmxl2*^loxP/-^; *Vasa-Cre*; *Amhr2*^*wt/Cre*^). These single and double conditional knockouts were studied at various postnatal stages.

Females of the different genotypes were fertile. The histological features of the ovary were also similar between females of the different genotypes (S6 Fig).

In males, fertility tests performed until the age of six months showed no significant differences between Sertoli cell cKO (9.1 ± 2.6 pups per litter), germ cell cKO (9.4 ± 3.4 pups per litter), Sertoli and germ cell dcKO (9.6 ± 2.9 pups per litter) and control *Dmxl2*^*loxP/loxP*^ (9.2 ±3.4 pups per litter) mice. In addition, histological analyses of testis sections from six-month-old mice of the various genotypes revealed no specific phenotype (S7A Fig), and sperm parameters were similar in Sertoli and germ cell dcKO and *Dmxl2*^*loxP/loxP*^ control mice (S7B Fig). *Dmxl2* expression increases greatly with the onset of spermatogenesis at puberty (Fig 5A). We therefore studied sperm parameters and testis differentiation at the end of the first wave of spermatogenesis.

### *Dmxl2* is required for the first wave of sperm production

**Fig 6:**
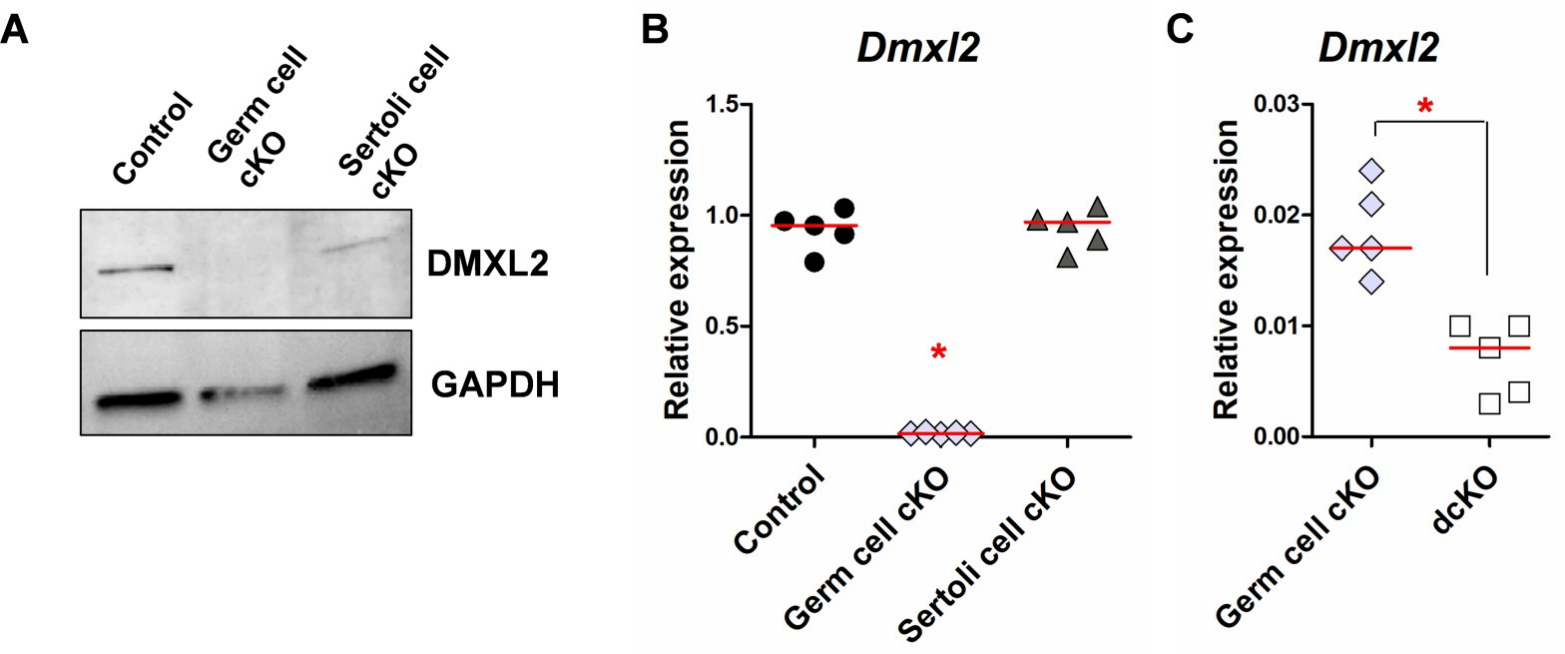
*Dmxl2*/DMXL2 levels in the testes of adults of different genotypes. **(A)** Western blot on control, germ cell cKO and Sertoli cell cKO testes, 6 months after birth. DMXL2 protein was detected in control and Sertoli cell cKO testes. **(B, C)** RT-qPCR analyses of *Dmxl2* transcript levels in the testes of mice of the four different genotypes (7 weeks after birth), with *Dmxl2* mRNA quantification based on the amplification of exon 7. *Dmxl2* transcript levels are much lower in germ cell cKO testes (B and C), but a complete abolition of the expression of this gene was observed only in dcKO testes (C). Asterisks indicate significant differences between genotypes (pValue<0.05).

**Fig 7:**
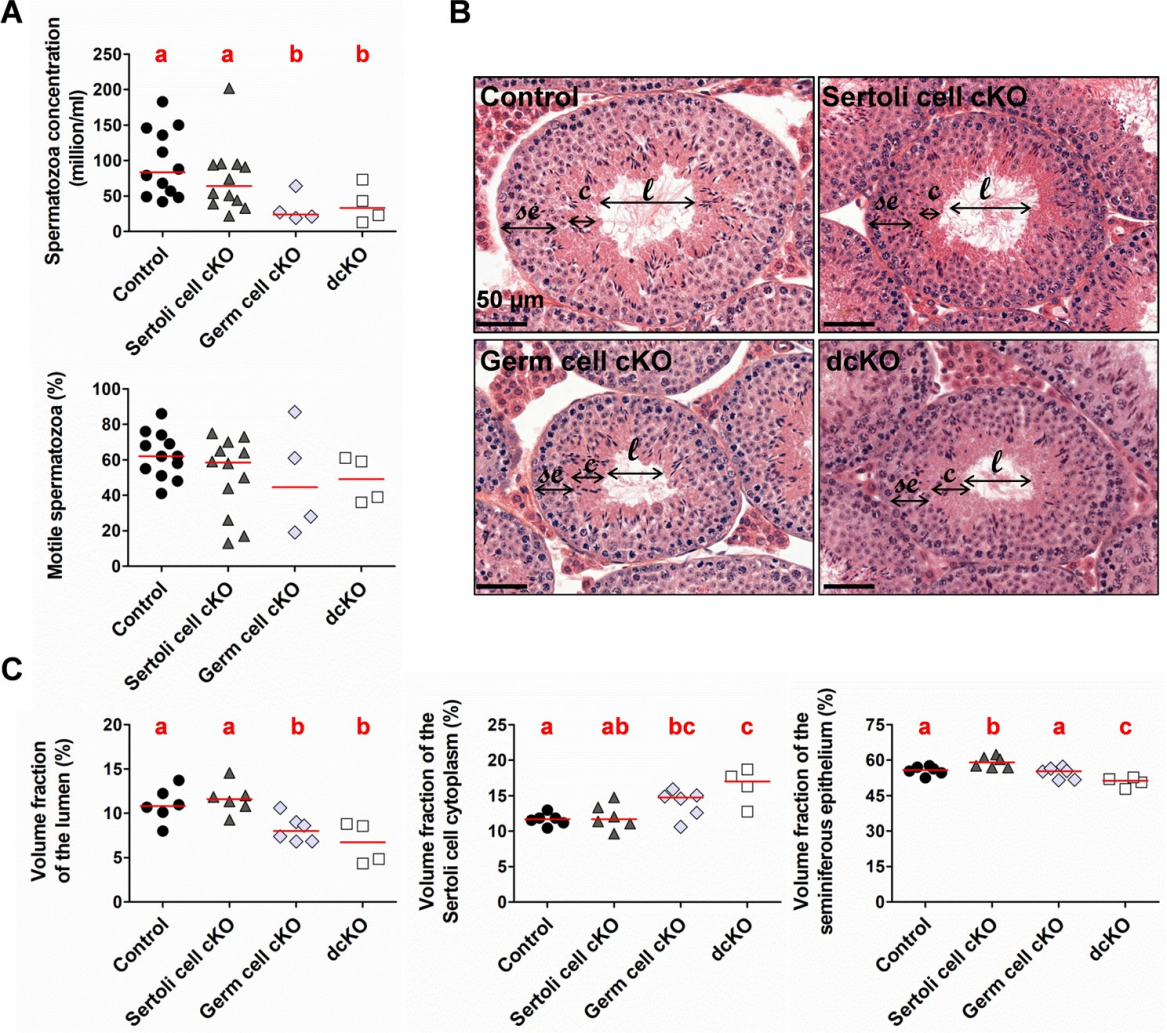
Testicular parameters and morphological appearance of testes displaying cell-specific *Dmxl2* KO from seven-week-old mice. **(A)** Sperm parameters of testes with a *Dmxl2* cell-specific KO. Epididymal sperm concentration in germ cell cKO (*Dmxl2*^loxP/-^; *Vasa-Cre)* testes was 71% lower than that in control testes (*Dmxl2*^*loxP/loxP*^) (pValue = 0.021), and that in Sertoli and germ cell dcKO (*Dmxl2*^loxP/-^; *Vasa-Cre*; *Amh-Cre)* testes was 61% lower than that in control testes (pValue = 0.035). No significant difference was detected between germ cell cKO and dcKO testes (pValue = 0.7429). The motility of the spermatozoa was unaffected. **(B)** Histological appearance and **(C)** stereological analyses of control, Sertoli cell cKO, germ cell cKO and Sertoli and germ cell dcKO (dcKO) testes. The fraction of the volume occupied by the Sertoli cell cytoplasm (c) in the lumen is 41.7% greater in *Dmxl2* dcKO testes than in control testes (pValue = 0.02), whereas the lumen area fraction (l) presents a mirror-image 36.4% decrease (pValue = 0.001). A similar effect was observed in comparisons of germ cell cKO and control testes (pValues of 0.02 and 0.03, respectively). Slight differences in seminiferous epithelium thickness (se) were observed in *Dmxl2* dcKO testes relative to control (pValue = 0.01). Significant differences are indicated by different letters.

We first assessed *Dmxl2*/DMXL2 transcript and protein levels in the testes of mice of the different genotypes. We found that the germ cells were the major site of *Dmxl2*/DMXL2 expression in adults (Fig 6A and 6B). Nevertheless, *Dmxl2* transcript detection was completely abolished only in *Dmxl2* dcKO testes (Fig 6C), which were therefore considered to display a testis-specific *Dmxl2* KO. The sperm parameters of control (*Dmxl2*^*loxP/loxP*^) (*n* = 12), Sertoli cell cKO (*Dmxl2*^*loxP/loxP*^; *Amh-Cre*) (*n* = 11), germ cell cKO (*Dmxl2*^loxP/-^; *Vasa-Cre*) (*n* = 4) and Sertoli and germ cell dcKO (*Dmxl2*^loxP/-^; *Vasa-Cre*; *Amh-Cre)* (*n* = 4) mice were analyzed seven weeks after birth (Fig 7A). Sperm concentration was more than 60% lower in mice with no *Dmxl2* expression anywhere in the testes (dcKO), and in mice lacking *Dmxl2* only in the germ line (germ cell cKO), despite a normal testis/body weight ratio (S4 Table). The percentage of motile sperm was similar in the four mouse lines, demonstrating that only the total number of spermatozoa was affected. Stereological analyses of germ cell cKO and dcKO testis sections revealed a significantly larger fraction occupied by the Sertoli cell cytoplasm in the center of the seminiferous tubules than in WT sections, whereas the lumen area was significantly smaller (Fig 7B and 7C; S8 Fig). In addition, the seminiferous epithelium occupied a smaller area in dcKO testes than in control (pValue = 0.001) or germ cell cKO testes (pValue = 0.05), suggesting that the number of germ cells was smaller.

**Fig 8:**
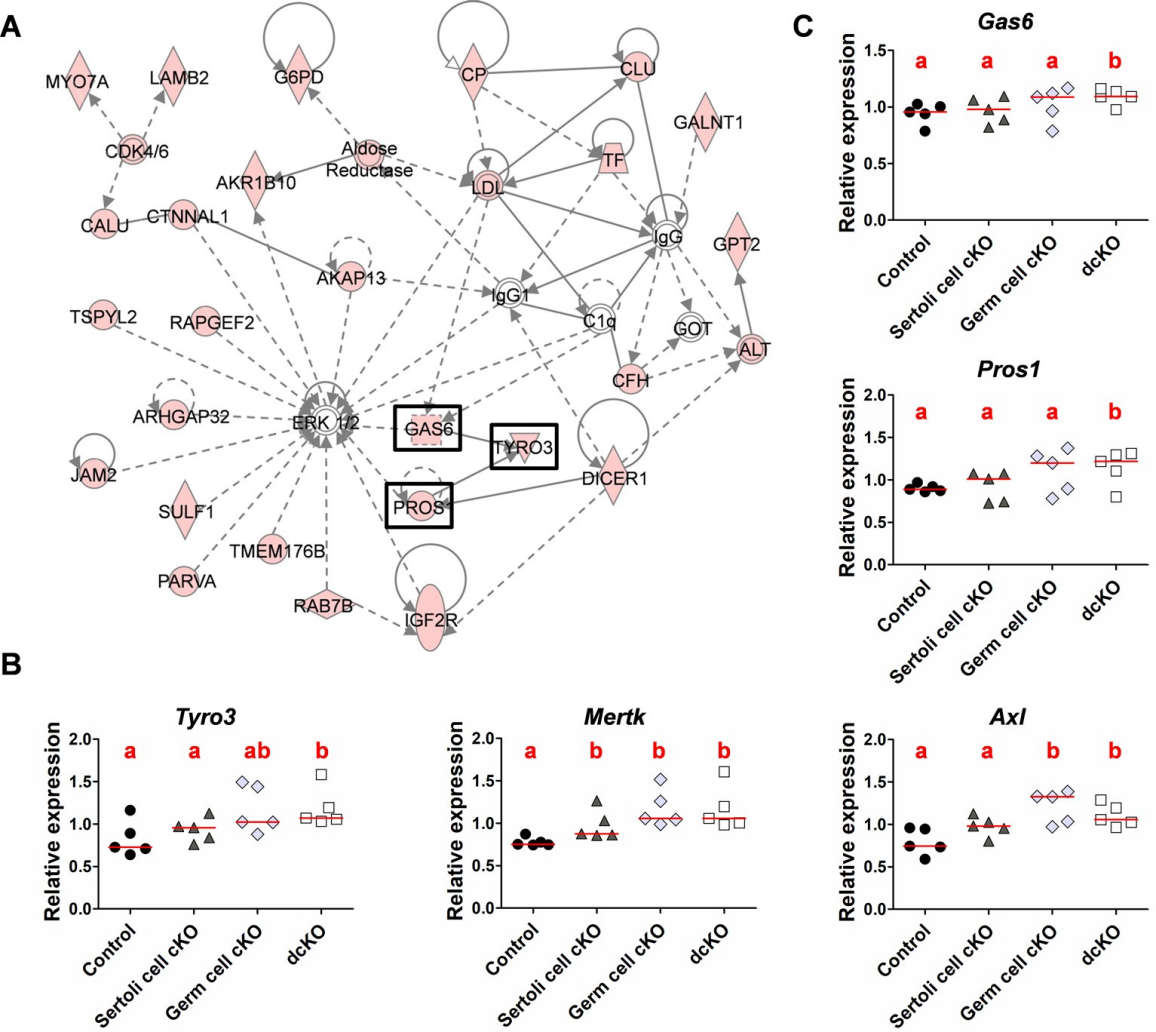
Upregulation of genes involved in phagocytosis in the absence of *Dmxl2* in testes from seven-week-old animals. **(A)** The top-ranking functional networks according to IPA. Thirty of the 161 genes upregulated in *Dmxl2* dcKO testes (molecules in red) are involved in ERK1/2 pathway regulation. The TAM receptor (TYRO3) and ligands (GAS6 and PROS1) are boxed. **(B, C)** RT-qPCR analyses of the expression of the three TAM receptors (*Tyro3*, *Axl*, *Mertk*) **(B)** and their ligands (*Gas6*, *Pros1*) **(C)** in the testes of mice of the four different genotypes (Control: *Dmxl2*^loxP/loxP^; Sertoli cell cKO: *Dmxl2*^*loxP/loxP*^; *Amh-Cre*; Germ cell cKO: *Dmxl2*^loxP/-^; *Vasa-Cre*; dcKO: *Dmxl2*^loxP/-^; *Vasa-Cre*; *Amh-Cre*). Different letters indicate significant differences between genotypes (pValue<0.05).

**Fig 9:**
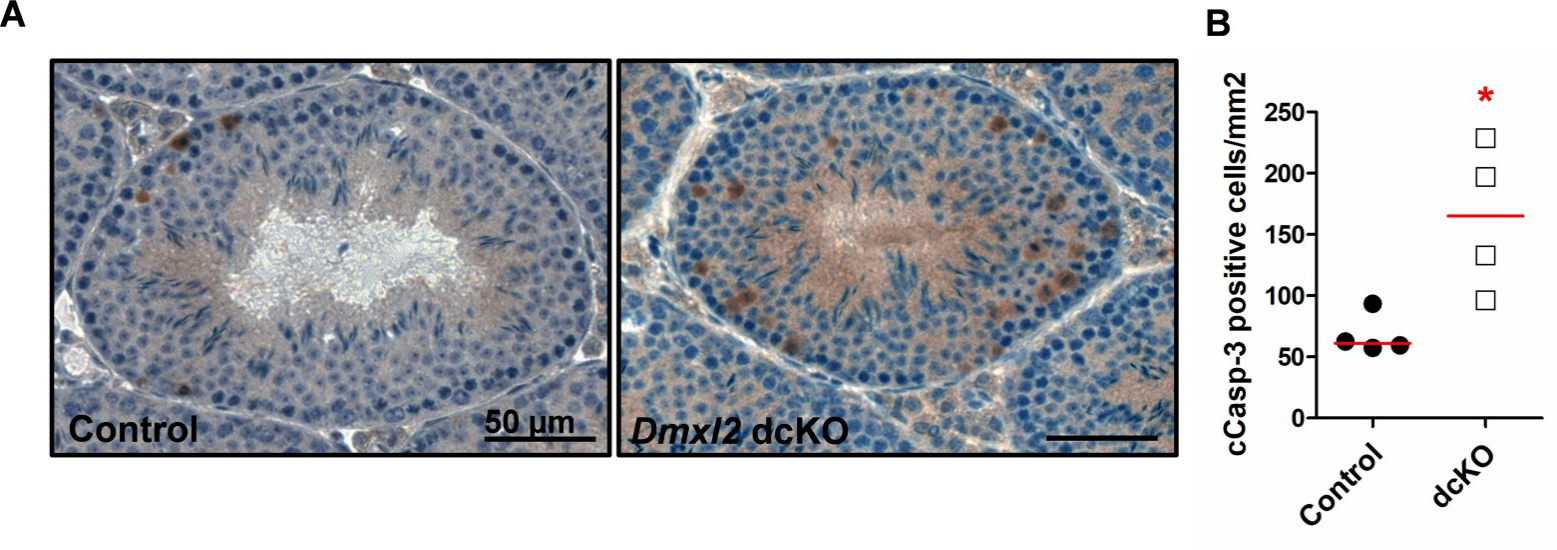
Higher levels of apoptosis in the absence of *Dmxl2* in testes from seven-week-old animals. (**A**) Immunohistochemistry was used to detect cCasp-3 (brown) in control and dcKO testes. **(B)** The cCasp-3-positive cells were counted in each genotype, and the results are expressed per mm^2^ of seminiferous tubules. Asterisks indicate statistical significance (pValue = 0.028).

We then used an RNA sequencing approach to characterize the molecular consequences of the absence of DMXL2 expression in testes from seven-week-old animals, comparing dcKO (*Dmxl2*^loxP/-^; *Vasa-Cre*; *Amh-Cre*) and *Dmxl2*^*loxP/loxP*^ control testes. RNA-sequencing identified 363 genes as differentially expressed in dcKO testes relative to control gonads: 161 genes were upregulated and 202 were downregulated (S1 File). We identified the cell types affected by *Dmxl2* loss–of-function in the testes, by assessing the cellular expression profiles of the 363 differentially expressed genes based on RNA-sequencing data obtained from the Gene Expression Omnibus http://www.ncbi.nlm.nih.gov/geo/; accession number GSE43717; [31]) (S1 File). According to these data, *Dmxl2* is weakly expressed in Sertoli cells and much more strongly expressed in the germ line, mostly in spermatogonia and spermatocytes, consistent with our findings (S1 File). The heat maps of the 161 upregulated genes (S9A Fig) and of the 202 downregulated genes (S9B Fig) were mirror images. Indeed, the genes upregulated in dcKO mice were mostly genes expressed by Sertoli cells and spermatogonia, whereas those downregulated were mostly genes expressed by spermatocytes and spermatids. Accordingly, the leading functional annotation for downregulated genes was “spermatogenesis” (Benjamini-Hochberg adjusted pValue = 5 x 10^−4^, DAVID6.8; S2 File). These observations, together with our previous stereological data, suggest a defect affecting the first wave of spermatogenesis, with smaller numbers of spermatocytes and spermatids produced in dcKO gonads. As the number of Sertoli cells conditions the number of spermatogenic cells, we determined the numbers of SOX9-positive cells (i.e. Sertoli cells) in dcKO and control testes (S10 Fig). No difference was found between the two genotypes. Additional *in silico* analyses on upregulated genes highlighted apoptosis (apoptosis signaling, pValue = 0.0035) and endocytosis (phagosome maturation, pValue = 0.0036; clathrin-mediated endocytosis signaling, pValue = 0.0123; macropinocytosis signaling, pValue = 0.0186) processes as canonical pathways significantly upregulated in dcKO gonads, which could be related to the decrease in the number of spermatogenic cells at this stage (Ingenuity Pathway Analysis software, Qiagen; S3 File). Furthermore, the ERK1/2 pathway, which has been implicated in apoptosis and phagocytosis [32], was the leading functional network detected, encompassing 30 of the 161 upregulated genes (almost 20% of the upregulated genes). Three of the five members of the TAM (Tyro3 Axl Merkt) regulation pathway were highlighted in this network [33]: one receptor *Tyro3* and two ligands: *Gas6* (*growth-arrest-specific 6*) and *Pros1* (*Protein S*) (Fig 8A). This pathway has been reported to be involved in the phagocytic activity of macrophages, but also in that of Sertoli cells, in which it plays a crucial role in ensuring fertility [34], [35]. Transcript levels for the five members of the TAM family (the three receptors, *Tyro3*, *Mertk*, *Axl*, and their two ligands, *Gas6*, *Pros1*) were analyzed in the testes of seven-week-old mice of the four genotypes (control, Sertoli cell cKO, germ cell cKO and dcKO) (Fig 8B). Interestingly, the transcript levels of all these genes were significantly higher in dcKO than in control testes (pValue <0.05), highlighting a general enhancement of the TAM regulatory pathway and, potentially, of Sertoli cell phagocytosis in the absence of DMXL2 expression in the testes. This increase in phagocytic activity may reflect higher levels of germ cell apoptosis in dcKO testes. Cleaved caspase-3 (cCasp-3) expression was detected by immunohistochemistry and cCasp-3-positive cells were counted in dcKO and control testes (Fig 9A and 9B respectively). The proportion of apoptotic cells (cCasp-3-postive cells/mm^2^) was significantly higher in dcKO testes (pValue = 0.02), despite variations within animals.

In conclusion, in the absence of *Dmxl2* expression in the testes, spermatogenesis appears to be less efficient, with higher levels of germ-cell apoptosis, whereas the phagocytic activity of Sertoli cells seems to be enhanced during the first wave of spermatogenesis.

## Discussion

We describe here, for the first time, the neonatal lethality of *Dmxl2* KO (*Dmxl2*^*tm1a/tm1a*^) in mice, with a maximum survival of 10 to 12 hours after birth. This observation contrasts with that of a previous study in which *Dmxl2*^*tm1a/tm1a*^ was reported to be lethal during fetal development [18]. We therefore introduced the *Dmxl2* tm1a mutation into two different genetic backgrounds, the C57Bl/6N and FVB/NRj strains, in which neonatal lethality kinetics were similar. Our first observations of *Dmxl2* KO pups revealed abnormal feeding behavior, with an absence of milk in the stomach. A complete inability to feed inevitably leaves to neonatal death, due to the absence of nourishment, and because the liquid derived from milk is essential for homeostatic processes in newborn [36]. Nevertheless, death linked to milk deprivation occurs within 12 to 24 h and therefore seems unlikely to be the sole cause of premature death in *Dmxl2* KO mice. Morphometric studies revealed no major defect in organogenesis, but signs of neurological and metabolic problems were observed.

The tissue-specific expression of *Dmxl2* has been analyzed during fetal and postnatal development. *Dmxl2* expression is strongly detected in the olfactory mucosa of the fetus, an area closely associated with migrating GnRH neurons [37]. DMXL2 has been reported to be crucial for the number, activation and maturation of GnRH neurons [18], [19]. It therefore seems likely that the GnRH neuron defects observed in adult *nes-Cre*; *Dmxl2* ^wt/loxP^ mice result from early fetal dysfunction. The transmission of olfactory information was impaired in *Dmxl2* KO neonates, which displayed very poor olfactory bulb neuron activation. As olfaction is required for suckling behavior in rodents [23], this neurological transmission defect in KO pups may contribute to the absence of feeding. However, this observation is insufficient to account for the premature death of these pups, and this specific example of neurological failure probably reflects more severe impairments, as observed in human patients, in whom *DMXL2* haploinsufficiency leads to polyneuropathy [18].

During gestation, fetal homeostasis is essentially managed by the placenta, whereas, after birth, the neonate must rapidly adapt to a stressful situation with new metabolic needs. A failure to establish energy homeostasis rapidly leads to the death of the neonate, over a period of time similar to that observed for *Dmxl2* KO mice. Indeed, death may occur within eight hours of birth in some cases of glucose homeostasis problems [38], or between 10 and 14 hours after birth if autophagy mechanisms are disrupted [39], [40]. Interestingly, neonatal *Dmxl2* KO pups present signs of metabolic/homeostasis problems, such as hypoglycemia in particular. During the first few hours after birth, the pup experiences a period of starvation during which gluconeogenesis is not fully active [41]. Glycogen is stored in the liver during fetal development, to prevent hypoglycemia in the neonate. Glycogen is an important source of glucose, which is released via glycogenolysis [42]. This adaptive mechanism is managed by a hormonal network, in which insulin levels decrease and the secretion of glucagon and glucocorticoids increases. DMXL2 was not detected in the pancreas or liver at birth, but was found in the adrenal glands, focusing attention on glucocorticoids and the possible effect of DMXL2 on their secretion. Intriguingly, hypoglycemia was observed only in male KO pups, highlighting the sex-specific nature of DMXL2 function in glucose homeostasis and, possibly, in adrenal sex-specific functions. However, as the timing of death was similar for both male and female KO pups, hypoglycemia cannot be the main event causing premature death. Autophagy is the first source of energy after birth, before efficient glycogenolysis is established. Autophagy levels are low during embryogenesis, but this process is upregulated in various tissues at birth (including the heart in particular) and is maintained at high levels from 3 to 12 hours after birth [39]. Autophagy eliminates aberrant or obsolete cellular structures/organelles. It is the primary means of degrading cytoplasmic constituents within lysosomes. Autophagy is also important for the cellular response to starvation, as the amino acids it generates can be used directly as a source of energy, or converted into glucose by the liver. Mice with deficiencies of ATG5 or ATG7 (autophagy-related proteins 5 and 7, respectively), which are involved in autophagosome formation, die within the first 12 hours of birth [39], [40], a timing very similar to that observed for *Dmxl2* KO pups. Many WD40 proteins have been implicated in autophagy: Atg18 (autophagy-related protein 18) [43], EPG-6 (ectopic PGL granules 6) [44], AMBRA1 (autophagy/beclin-1 regulator 1) [45], ALFY (autophagy‐linked FYVE protein) [46] and WDR47 (WD40-repeat 47) [47]. A possible new function of the DMXL2 protein in autophagy should, therefore, be investigated, as it might explain the premature death of *Dmxl2* knockout mice.

In addition to its role in neurological or homeostatic processes, DMXL2 was also thought to be associated with reproductive functions. Indeed, *Dmxl2* is expressed in the germ cells and supporting cells of the gonads of both sexes, with a timing during development suggesting involvement in the major events of ovary and testis differentiation.

In the ovary, *Dmxl2* expression increases after the onset of meiosis (E14.5) to reach a peak at birth. This period corresponds to germ-cell nest formation, breakdown and primordial follicle formation. However, the histological features of the ovaries were identical in *Dmxl2* KO mice and controls at birth, as some primordial follicles were already visible in the gonads of both genotypes. Furthermore, cell-specific *Dmxl2* knockout did not result in any fertility problems or ovarian abnormalities in adult females. Analyses of gene expression in *Dmxl2* KO gonads at P0 revealed molecular disorders in the ovaries, which seemed to be under stress and trying to adapt to the loss-of-function of *Dmxl2*, possibly by increasing the expression of other WD40 protein-encoding genes (i.e. *Coro2b* and *Fbxw8*). More interestingly, the *Aph1b* gene was found to be upregulated in the gonads of *Dmxl2* KO mice of both sexes. *Aph1b* encodes one of the four subunits of the γ-secretase complex, which plays a key role in the Notch signaling pathway, and this subunit is involved in the stability of the complex [48], [49], [50]. DMXL2 has been implicated in Notch signaling, in which it controls the V-ATPase pumps responsible for regulating the pH of the endocytic vesicles in which γ-secretase acts [51], [52], [13], [14]. In *Drosophila* ovaries, DMXL2/Rbcn-3α has even been shown to be involved in follicle formation, through its control of the Notch pathway [7]. The Notch pathway plays an important role in folliculogenesis that has been conserved during evolution, from flies to mammals [7], [53], [54]. Nevertheless, we show here that DMXL2 is not crucial for Notch signaling in mouse ovaries. The potential decrease in γ-secretase activity in ovaries lacking *Dmxl2* is probably counterbalanced by an increase in the stability of the complex (via *Aph1b* upregulation). The important role of DMXL2/Rbcn-3α in folliculogenesis and female fertility is, therefore, not conserved in mice.

In testes, only a few other genes in addition to *Aph1b* were deregulated at P0 in *Dmxl2* KO gonads. Nevertheless, *Dmxl2* expression in the testes began to increase between P5 and P15, coinciding with spermatogenesis, suggesting a role in postnatal gametogenesis rather than early testis differentiation. Consistent with this hypothesis, histological/stereological observations and analyses of sperm parameters in the gonads of mice with a testis-specific *Dmxl2* KO (Sertoli and germ cell dcKO) revealed a disruption of the first spermatogenic wave, resulting in a sperm concentration 60% lower than that in the controls. The seminiferous tubules presented an expended Sertoli cell cytoplasm, with a shorter lumen, suggesting higher levels of phagocytosis by the supporting cells. Transcriptomic and immunohistochemical analyses confirmed these observations, highlighting a decrease in the spermatocyte/spermatid fraction and an increase in apoptosis, accompanied by an increase in the levels of phagocytosis regulators. In particular, the TAM pathway was found to be upregulated in the absence of *Dmxl2* expression. Three of the TAM proteins belong to the receptor protein tyrosine kinase (RPTK): TYRO 3, AXL and MERTK. Two related proteins, GAS 6 and Protein S (*Pros1*), act as their ligands. These five TAM proteins are expressed by Sertoli cells in the testes [34]. Males lacking the three TAM receptors (TAM^-/-^) are sterile due to an impairment of the phagocytic function of Sertoli cells, which is essential for the elimination of apoptotic germ cells [34], [35], [55]. TAM receptor dimers bind their two ligands, which in turn bind to the phosphatidylserine exposed at the surface of apoptotic cells [56]. In this study, germ cell apoptosis rates were significantly higher in the absence of DMXL2 (dcKO testes), suggesting that the TAM regulation (and thus, probably, the phagocytic activity of Sertoli cells) is enhanced in mutants due to germ cell dysfunction. Mice without DMXL2 expression in the germ line had a phenotype similar to that of dcKO mutants, with low sperm concentrations at puberty (Fig 7A). Together, these results suggest that DMXL2 exerts its principal function in germ cells, during the meiotic process occurring at the onset of spermatogenesis. Changes in its expression may affect germ cell differentiation, with higher rates of apoptosis and phagocytosis by Sertoli cells clearing abnormal spermatocytes/spermatids from the seminiferous tubules and resulting in a lower sperm concentration. Nevertheless, sperm production normalized at later stages of testis development, indicating that the functions of DMXL2 are essentially limited to the first wave of spermatogenesis or that compensatory processes occur after puberty. As suggested by Busada et al. for *Rhox13* [57], *Dmxl2* expression in spermatogenic cells may be advantageous in mice, supporting early fertility by providing additional germ cells at the start of the animal’s reproductive life.

## Materials and methods

### Mice

Animals were handled in accordance with the guidelines on the Care and Use of Agricultural Animals in Agricultural Research and Teaching (Authorization no. 91–649 for the Principal Investigator, and national authorizations for all investigators). The protocol was approved by the Ethics Committee for Animal Experiments of the Jouy-en-Josas Institute and AgroParisTech (Permit Number: 12/184). *Dmxl2*^*tm1a(EUCOMM)Wtsi*^ (*Dmxl2*^*wt/tm1a*^) mice with a C57Bl/6N genetic background were provided by the Wellcome Trust Sanger Institute (International Mouse Phenotype Consortium (IMPC): https://www.mousephenotype.org/data/genes/MGI:2444630). The mutation corresponded to a knock-in of the targeting vector between *Dmxl2* exons 6 and 10 (see Fig 1) [20], [21], [22]. We backcrossed *Dmxl2* mutant mice onto the FVB/NRj strain (JANVIER Laboratories) for 10 generations. *Dmxl2*^*wt/tm1a*^ mice with this genetic background were crossed to generate *Dmxl2*^*wt/wt*^ *(*wild-type, WT), *Dmxl2*^*wt/tm1a*^ (heterozygous, HTZ) and *Dmxl2*^*tm1a/tm1a*^ (knocked-out, KO) mice.

Conditional knockouts of *Dmxl2* were obtained by crossing *Dmxl2*^*wt/tm1a*^ mice with FlpO (FLP) recombinase-expressing mice (*Rosa26-FlpO*) [27] to remove the β-galactosidase cassette and the neomycin resistance gene and to create *Dmxl2*^*loxP/loxP*^ mice (in which the *Dmxl2* exon 7 is floxed, see Fig 1A). *Dmxl2* conditional knockout in germ cells was achieved by crossing *Dmxl2*^*loxP/loxP*^ mice with *Vasa-Cre* mice (FVB-Tg(Ddx4-cre)1Dcas/J) [28] to obtain *Dmxl2*^*wt/-*^ *; Vasa-Cre* mice in the F1 generation. Due to the mode of *Vasa-Cre* transmission, only F1 males (*Dmxl2*^*wt/-*^ *; Vasa-Cre)* of less than eight weeks of age were then used to generate *Dmxl2*^*loxP/-*^ *; Vasa-Cre* F2 mice (germ cell cKO).

The conditional knockout of *Dmxl2* in granulosa cells was achieved by crossing *Dmxl2*^*loxP/loxP*^ mice with *Amhr2*-Cre mice (*Amhr2*^*wt/Cre*^) [30] to obtain *Dmxl2*^*wt/loxP*^; *Amhr2*^*wt/Cre*^ F1 mice. We then crossed *Dmxl2*^*loxP/loxP*^ mice with *Dmxl2*^*wt/loxP*^ *; Amhr2*^*wt/Cre*^ mice to obtain *Dmxl2*^*loxP/loxP*^ *; Amhr2*^*wt/Cre*^ F2 mice (granulosa cell cKO).

*Dmxl2* was knocked out specifically in Sertoli cells by crossing *Dmxl2*^*loxP/loxP*^ mice with *Amh-Cre* mice [29]. The *Dmxl2*^*wt/loxP*^; *Amh-Cre* F1 mice were then crossed with each other to generate *Dmxl2*^*loxP/loxP*^ *; Amh-Cre F2 mice* (Sertoli cell cKO). We also generated double conditional mutant mice (dcKO) by crossing young *Dmxl2*^*loxP/-*^; *Vasa-Cre* males (six to eight weeks of age) with either *Dmxl2*^*loxP/loxP*^ *; Amh-Cre* or *Dmxl2*^*loxP/loxP*^; *Amhr2*^*wt/Cre*^ females to produce *Dmxl2*^*loxP/-*^; *Vasa-Cre; Amh-Cre* males (Sertoli and germ cell *Dmxl2* dcKO) or *Dmxl2*^*loxP/-*^; *Vasa-Cre*; *Amhr2*^*wt/Cre*^ females (granulosa and germ cell *Dmxl2* dcKO), respectively.

Mice were housed under a 12 h light/12 h dark cycle at the UE0907 (INRA, Jouy-en-Josas, France), with *ad libitum* access to food.

### Mouse genotyping

Genomic DNA was obtained from tail biopsy specimens with the Kapa Express Extract kit (Kapa Biosystems), according to the manufacturer’s instructions. Total-knockout animals were genotyped by PCR amplification of the *Dmxl2* wild-type and tm1a *LacZ* alleles. Conditional-knockout animals were genotyped by the PCR amplification of *Dmxl2* exon 7, and the *Vasa-Cre*, *Amhr2-Cre* and *Amh-Cre* alleles (see S1 Table for primer sequences and Fig 1 for the location of *Dmxl2* primers). PCR was performed with the KAPA2G Fast Genotyping Mix, according to the manufacturer’s instructions (Kapa Biosystems).

### X-gal staining

For E14.5 embryos, maternal uterine horns were dissected out and transferred to cold 1 X PBS (Eurobio) for storage. The embryonic sacs were removed and used for genotyping. Embryos were rapidly rinsed in PBS, and fixed by incubation for 2.5 hours in a fixative solution containing 2% formaldehyde and 0.2% glutaraldehyde in PBS. The embryos were washed twice, for 30 minutes each, in PBS, and stained by overnight incubation at room temperature, in the dark, in 5 mM ferrocyanide, 5 mM ferricyanide, 20 mM MgCl_2_, 1 mg/ml X-gal (GX12836, Genaxis), 0.02% NP-40, 0.01% sodium deoxycholate, 20 mM Tris HCl pH 7.4. The following day, they were briefly rinsed and incubated in PBS for 30 minutes, before final fixation by incubation overnight at 4°C in 4% formaldehyde. The fixed embryos were rinsed in PBS and cleared as described by Schatz and coworkers [58].

For whole-mount staining, freshly dissected tissues (skinned heads, heart and lungs, digestive system (from the stomach to the large intestine), urogenital tracts, and skeletal muscle) from P0 animals were washed for 10 min in PBS supplemented with 0.01% Tween-20 (PBS-T), fixed by incubation for 10 min in 4% paraformaldehyde (PFA), and then subjected to two more washes in PBS-T, for 10 minutes each. For the brain and kidneys, pups were perfused with 4% PFA for 10 minutes and washed by incubation in PBS overnight at 4°C. All tissues were then stained by overnight incubation in 5 mM ferrocyanide, 5 mM ferricyanide, 4 mM MgCl_2_, 0.1% Triton X-100, 1 mg/ml X-gal, at 32°C, in a water bath. Tissues were washed for 10 min in PBS-T and then fixed again by incubation with 4% PFA overnight at 4°C. They were stored in 100% ethanol until imaging with a Leica M80 dissecting microscope fitted with a Leica DFC420 digital camera.

### Protein extraction and western blotting

Tissues from newborn WT or *Dmxl2*^tm1a/tm1a^ (*Dmxl2* KO) mice (brain, heart, liver, pancreas, kidneys, adrenal glands, testes and ovaries), or from adult WT mice (brain, heart, pancreas, kidneys, adrenal glands, lungs, liver, spleen, skeletal muscle, testes, epididymides, seminal vesicles, ovaries, uterus and mammary glands) were collected and snap-frozen in liquid nitrogen. For protein extraction, tissues were ground on dry ice, and transferred to a Dounce homogenizer, in which they were lysed in radioimmunoprecipitation assay (RIPA) buffer supplemented with protease inhibitors (Roche). Lysates were centrifuged for 20 min at 4°C and 16,000x*g*, supernatants were collected and the amount of protein present was determined by the Bradford method.

For each tissue, we subjected 25 μg of protein diluted in Laemmli buffer to electrophoresis in 4–15% Mini-PROTEAN TGX gels (Cat. 456–108310, Bio-Rad). Stained proteins of known molecular weight (range: 31–460 kD, Cat. LC5699, Invitrogen) were run simultaneously as standards. The bands obtained on electrophoresis were transferred onto a polyvinylidene difluoride membrane (Hybond-P PVDF; Amersham). The membrane was blocked by incubation in phosphate-buffered saline containing 1/1000 Tween-20 (PBS-T; Prolabo, France) supplemented with 4% (w/v) nonfat dried milk, and was incubated overnight at 4°C with primary antibody (anti-DMXL2 or anti-GAPDH; refer to S5 Table for a list of the antibodies used and the conditions in which they were used) diluted in PBS-T supplemented with 4% (w/v) nonfat dried milk. The blot was then washed three times with PBS-T, incubated for 45 min in PBS-T supplemented with 4% (w/v) nonfat dried milk plus the peroxidase-conjugated secondary antibodies, and washed thoroughly in PBS-T. Peroxidase activity was detected with the ECL-Plus detection system for western blots, according to the manufacturer’s instructions (Amersham). Immunoreaction signals were analyzed with an image analysis system (Advanced Image Data Analyzer software, LAS 1000 camera, Fujifilm).

### Electro-olfactogram recordings

Electro-olfactogram (EOG) recordings were made on the olfactory mucosa in an opened nasal cavity configuration, on hemi-heads of newborn HTZ and KO mice, as previously described [59]. The hemi-head was kept under a constant flow of humidified filtered air (1000 ml/min) delivered close to the septum through a 9 mm glass tube. This tube was positioned 2 cm from the epithelial surface. The olfactory system was stimulated by blowing air puffs (200 ms, 200 ml/min) through an exchangeable Pasteur pipette containing a filter paper impregnated with 20 μl of the odorant, enclosed in the glass tube. The odorants used were diluted in mineral oil (hexanal from 1:10000 to 1:10; limonene from 1:1000 to 1:10 and carvone at 1:100). EOGs were recorded at two separate centrally located positions on turbinates IIb and IIa. EOG signals were analyzed and peak amplitudes were measured with a Clampfit 9.2 (Molecular Devices). Values were averaged for each set of conditions. Means ± SEM were plotted with GraphPad, and statistical analyses were performed with a Fisher-Pitman two-sample exact permutation test (R software using the Rcmdr.Plugin.Coin package (pValue<0.05)) to compare the response between HTZ and KO animals for a given concentration of odorant.

### Exposure to odorant for c-Fos detection in the olfactory bulb

Newborn pups were isolated from their dams and placed in a new cage for 30 min in a quiet room. They were then exposed, for 10 minutes, to odorants in a tea ball containing filter paper impregnated with 20 μl of a mixture of 12 odorants (equimolar mixture of anisole, citral, heptanal, isoamyl acetate, lyral, lilial, octanol,1-4-cineol, isomenthone, limonene, carvone, and pyridine diluted to a final concentration of 10^-3^M). Pups were killed 60 min after the end of the exposure period. Heads were skinned, and prepared as described in the “Immunohistochemistry” paragraph for c-Fos immunodetection. For all coronal olfactory bulb sections, we took four dorsal and ventral images. Images were acquired blind to treatment, at a magnification of x100. They were analyzed with ImageJ (Rasband, W.S., ImageJ, U.S. National Institutes of Health, Bethesda, Maryland, USA, http://imagej.nih.gov/ij/) for the thresholding of specific c-Fos staining, as previously described [60]. The area of the olfactory bulb was measured after DAPI staining, for quantification of the proportion of the plexiform extern and the glomerular layer area displaying c-Fos staining. The same threshold was applied to all images from the same experiment on the same litter. DAPI staining in each area was also quantified, for the evaluation of neuron density. Median values were plotted with GraphPad, and statistical analyses were performed with Fisher-Pitman two-sample exact permutation tests (R software, using the Rcmdr.Plugin.Coin package (pValue<0.05)) to compare HTZ and KO animals for c-fos activation and neuron density.

### Measurement of blood glucose and plasma insulin concentrations

Glucose concentrations were determined in blood samples from newborn pups on P0, with FreeStyle Optium Xceed Blood Glucose meters (Abbott). Glucose concentrations were determined after three hours of starvation (separation of the pup from its dam).

Insulin plasma concentrations were determined for each pup, with the Mouse Ultrasensitive Insulin ELISA kit, according to the manufacturer’s instructions (Alpco).

Median values were plotted with GraphPad, and statistical analyses were performed with Fisher-Pitman two-sample exact permutation tests (R software, using the Rcmdr.Plugin.Coin package (pValue<0.05)) to compare male and female pups of each genotype.

### Expression analysis by RT-qPCR

We studied *Dmxl2* expression during gonad development, by extracting total RNA from pools of ovaries or testes at different developmental stages, with the RNeasy Mini or Micro kit (Qiagen), depending on the amount of tissue. Three biological replicates were prepared for each stage and sex. The Maxima First-Strand cDNA Synthesis Kit (Thermo Scientific) was used to synthesize cDNA for RT-qPCR from 200 ng of RNA. RT-qPCR was performed in triplicate for all genes with the Absolute Blue SYBR Green ROX mix (Thermo Scientific), in the StepOnePlus Real-Time PCR System (Applied Biosystems). Based on the output of the GeNorm program, we used *ActB*, and *Ywhaz* as the reference genes for this study (S1 Table). The results were analyzed with qBase Software [61].

### Immunohistochemistry

Dissected tissues were fixed in by incubation in 4% PFA in PBS at 4°C for 2 hours (P0 and P5 gonads), overnight (P28 gonads) or for 24 hours (skinned heads of neonates). They were then cryoprotected with various concentrations of sucrose in PBS (0, 12%, 15%, and 18% for gonads, or 0, and 30% for heads). Tissues were finally embedded in Tissue-Tek O.C.T. Compound (Sakura Finetek Japan) and frozen at -80°C. Cryosections (7 μm for gonads, 20 μm for coronal head specimens) were cut and stored at -80°C. Sections were air-dried, rehydrated in PBS and permeabilized by incubation with 0.5% Triton, 1% BSA in PBS for 30 minutes. The tissue sections were then incubated with the primary antibodies (listed in S5 Table) overnight at 4°C (2 days for c-Fos). Sections were washed several times in PBS and then incubated with secondary antibodies for 45 min at room temperature (overnight for c-Fos). Finally, slides were rinsed in PBS, mounted in Vectashield mounting medium with DAPI (Vector) and images were acquired with a DP50 CCD camera (Olympus).

### Microarray analyses

Freshly dissected P0 ovaries and testes were snap-frozen in liquid nitrogen. Three independent total RNA extractions were performed on pools of WT and KO ovaries and testes, with the RNeasy Mini (for testes) or Micro kit (for ovaries) (Qiagen). RNA quality was checked with an Agilent Bioanalyzer and 200 ng of total RNA for each set of conditions was hybridized with a Mouse WG-6 v2.0 Expression BeadChip (Illumina) (Pitié-Salpêtrière Postgenomics Platform–P3S, http://www.p3s.chups.jussieu.fr, Paris, France). Raw data were corrected for background by the “normexp” method, and quantile-normalized with the Limma package, through Bioconductor in the R statistical environment (version 2.15.0). Raw pValues were adjusted by the Benjamini-Hochberg method (false discovery rate) [62]. The quality of the expression data was checked by generating boxplots for raw expression data, density plots for normalized data, and by producing scatter plots and calculating Pearson’s coefficient for the correlation between arrays, with the Ringo package. The microarray data were assigned Gene Expression Omnibus number GSE115194 and are publicly available (https://www.ncbi.nlm.nih.gov/geo/query/acc.cgi?acc=GSE115194). RT-qPCR validations were performed as previously described, on three independent pools of gonads per genotype (XX and XY, WT versus KO) and the results were normalized against two housekeeping genes (*ActB* and *Ywhaz*, according to GeNorm analyses) with qBase Software. Means ± SD were plotted with Excel, and statistical analyses were performed by ANOVA followed by Fisher’s LSD test in InVivoStat software [63].

### Evaluation of mouse fertility

Six-week-old male and female conditional mutant mice were paired with wild-type FVBN mice for a period of six months. Breeding cages were monitored daily and gestations, birth dates and litter sizes were recorded. At the end of the breeding trial, the gonads and epididymides were harvested and either snap frozen for molecular analysis or fixed for histological analysis.

The evaluation of male fertility was completed by the use of the IVOS I CASA system (Computer Assisted Sperm Analysis, at Hamilton Thorne Inc., Beverly, MA, USA) to assess semen motility at the ages of seven weeks and six months. The cauda epididymis was plunged into 100 μl of TCF buffer (Tris, citrate and fructose buffer) and swimming spermatozoa were collected after incubation for 30 minutes at 37°C. A 4 μl aliquot was placed in a standardized four-chamber Leja counting slide (Leja Products B.V., Nieuw-Vennep, the Netherlands). Ten microscope fields were analyzed on an automated stage, using the predetermined starting position within each chamber. Statistical analyses were performed with the mean values for these ten fields, for at least 500 cells. Each sample was analyzed twice (two different Leja wells). In total, 30 frames were captured at 60 frames/s, with software settings as follows: cell detection with a minimum contrast of 50, a minimum cell size of 4 pixels, and a cell intensity of 80; the cutoff value for progressive cells was 50 μm/s for VAP and 80.0% for STR. Slow cells were considered to be static and had a VAP cutoff of 7.4 μm/s and a VSL cutoff of 6.6 μm/s. Median values were plotted with GraphPad, and statistical analyses were performed with Fisher-Pitman two-sample exact permutation tests (R software, using the Rcmdr.Plugin.Coin package (pValue<0.05)) to compare the sperm parameters of *Dmxl2*^*loxP/loxP*^ males and the various conditional mutants.

### Histological, stereological and immunohistochemical analyses

The dissected gonads were fixed in Bouin’s solution for 2 hours at room temperature for P0 gonads and overnight at 4°C for adult gonads. They were washed several times in 70% ethanol and then dehydrated in a series of solutions of increasing concentrations of ethanol (90%, 100%) and butanol (50%, 100%). The tissues were then embedded in paraffin and 5 μm-thick sections were cut. Hematoxylin and eosin staining (HE staining) was performed by standard protocols for studies of tissue morphology. Images were captured with a Pannoramic Scan II (3DHISTECH) digital slide scanner.

For males, adult testis sections from control (*n* = 6), Sertoli cell cKO (*n* = 6), germ cell cKO (*n* = 6) and double conditional KO animals (*n* = 4) were analyzed in more detail. The volume fractions of the lumen, the residual Sertoli cell cytoplasm and the seminiferous epithelium were estimated on 200 seminiferous tubules per sample with the P2 grid of Appendix B of the chapter 4 of “Unbiased Stereology” [64, 65]. For each experiment, medians values were plotted with GraphPad, and statistical analyses were performed with Fisher-Pitman two-sample exact permutation tests in R software, with the Rcmdr.Plugin.Coin package (pValue<0.05).

For immunohistochemical analysis, Bouin’s solution-fixed testis sections (5 μm) from seven-week-old mice (control and *dmxl2* dcKO) were deparaffinized and subjected to antigen retrieval by heating in 0.01 M citrate buffer, pH 6.0 in a pressure cooker for 5 minutes. The sections were then incubated for 10 min in H_2_O_2_ (0.3%) and then for 30 min in a blocking and permeabilization buffer (PBS/1% BSA/0.5% Triton). The sections were incubated overnight at 4°C with primary antibodies (anti-cCasp3 and anti-SOX9 antibodies [66]; see S5 Table for the list of antibodies and dilutions used). The slides were then washed in PBS and incubated with a biotinylated anti-rabbit IgG for 45 minutes at room temperature. The primary antibody was omitted as a negative control. Antibody binding was detected with a Vectastain ABC kit (Vector Laboratories, PK-6100), and sections were counterstained with hematoxylin. Images were captured with a Pannoramic Scan II (3DHISTECH) digital slide scanner. Cleaved caspase-3-positive cells were manually counted on virtual slides obtained with the Pannoramic viewer (3DHISTECH software). Seminiferous tubules were outlined manually and their surface area was obtained by the Pannoramic viewer. The total number of cCasp-3-positive or SOX9-positive cells was divided by total seminiferous tubule surface area (μm^2^) and multiplied by 1,000,000 to obtain a number of positive cells/mm^2^. Cell counting was performed on 10 (for SOX9) or 30 (for cCasp-3) round seminiferous tubules (transverse sections) on testes from 4 different animals of the control and *Dmxl2* dcKO genotypes. Median values were plotted with GraphPad, and statistical analyses were performed with Fisher-Pitman two-sample exact permutation tests (R software, using the Rcmdr.Plugin.Coin package (pValue<0.05)).

### RNA sequencing

Total RNA was extracted from the testes of seven-week-old *Dmxl2*^*loxP/loxP*^ (WT mice) (*n* = 3) and *Dmxl2*^*loxP/-*^; *Amh-Cre*; *Vasa-Cre* (*n* = 3) mice with the RNeasy Mini kit (Qiagen). RNA quality was checked with an Agilent Bioanalyzer and 1 μg of total RNA from each sample was sent to the High-throughput Sequencing Platform of I2BC (Gif-sur-Yvette, Université Paris-Saclay, France) for oriented library preparation and sequencing. At least, 50 million 75 nt reads were generated per sample (SRA accession: SRP149657). Sequence libraries were aligned with the Ensembl 89 genome, with STAR [67], and gene table counts were obtained by applying RSEM to these alignments [68]. Statistical analyses of differential transcript accumulation were performed with R version 3.0.0 (R Development Core Team, 2013) with the Bioconductor package DESeq2 version 1.0.19 [69]. Fold-changes in expression were estimated by an empirical Bayes shrinkage procedure, which attenuated the broad spread of fold-change values for genes with low counts with negligible effects on genes with high counts [69]. The pValues were adjusted for multiple testing by the Benjamini and Hochberg method [62], and those with an adjusted pValue ≤0.05 were considered to be significant (S1 File).

RNA-sequencing data providing information about the gene expression profiles of different testis cell types were obtained from the Gene Expression Omnibus (accession number GSE43717; [31]). FPKM files containing normalized RNA‐Seq data for purified Sertoli cells (GSM1069639), spermatogonia (GSM1069640), spermatocytes (GSM1069641), spermatids (GSM1069642) and spermatozoa (GSM1069643) were compiled and data concerning the genes differentially expressed in t*Dmx2* KO testes were extracted (S1 File). FPKM values were log_2_-transformed to produce heat maps (pheatmap: Pretty Heatmaps. R package version 1.0.8; Raivo Kolde (2015); https://CRAN.R-project.org/package=pheatmap).

RT-qPCR validations were performed as previously described, with total testis RNA extracted from five animals per genotype (seven weeks of age), and results were normalized against three housekeeping genes *(ActB*, *Ywhaz* and *H2afz* (S1 Table), selected on the basis of GeNorm analyses) with qBase Software. For each experiment, median values were plotted with GraphPad, and statistical analyses were performed with Fisher-Pitman two-sample exact permutation tests in R software (Rcmdr.Plugin.Coin package (pValue<0.05)).

## Supporting information

S1 Table List of primers. (DOCX)

S2 Table List of genes deregulated in *Dmxl2* KO ovaries at birth (adjusted pValue <0.1). We found that 51 genes were differentially regulated between KO and WT ovaries (ordered according to their fold-change in expression): 28 were downregulated in KO ovaries, whereas 23 were upregulated. ^a^ Two of the upregulated genes were represented by several probes (*B3gat1* and *Tpm1*).
(DOCX)

S3 Table List of genes deregulated in *Dmxl2* KO testes at birth (adjusted pValue < 0.1). We found that 12 genes were differentially regulated between KO and WT testes (ordered according to their fold-change in expression). Three were downregulated and the other nine were upregulated in KO testes.
(DOCX)

S4 Table Weight of the testes and body weight in mice of the different genotypes at seven weeks after birth. (DOCX)

S5 Table List of antibodies and the conditions in which they were used. (DOCX)

S1 File RNAseq data from *Dmxl2* dcKO and *Dmxl2* ^loxP/loxP^ control testes and DE (differential expression) results. (XLSX)

S2 File DAVID clusters for DEseq data (adjusted pValue<0.05). (XLSX)

S3 File IPA pathways and networks obtained from DEseq data (adjusted pValue<0.05). (XLS)

S4 File Numerical data used to generate graphs. These data were those used to generate graphs and to perform statistical analyses in Figs 4A, 4D, 5A, 6B, 7A, 7C, 8B and 8C.
(XLSX)

S5 File Numerical data used to generate graph in Fig 9B. (XLSX)

S6 File Numerical data used to generate graph in S10 Fig. (XLS)

S1 Fig Glycemia and insulinemia at P0. **(A)** Blood glucose concentration at P0. Only *Dmxl2* KO XY pups (males) are hypoglycemic (20 mg/dl), with significantly lower (66.7% lower) blood glucose concentrations than WT pups (60 mg/dL). **(B)** Plasma insulin concentration at P0. Insulinemia was similar in *Dmxl2* WT, HTZ and KO pups (0.25 ng/ml). Significant differences are indicated by asterisks.
(TIF)

S2 Fig Specificity of the anti-DMXL2 antibody. At P0, DMXL2 was detected in the cytoplasm of germ cells and somatic cells in WT ovaries and testes. No staining was observed in *Dmxl2* KO gonads other than a faint background in male germ cells.
(TIF)

S3 Fig Histological appearance of *Dmxl2* KO gonads at birth. Hematoxylin and eosin staining revealed no obvious differences between *Dmxl2* KO and control gonads at birth, in terms of size and organization. The ovaries had germ cell nests in the cortex, and seminiferous cords were evident in the testes.
(TIF)

S4 Fig Morphological appearance of the ovaries of *Dmxl2* KO mice at birth. Immunofluorescence studies were performed with a germ cell marker (VASA, cytoplasmic staining) and a pre-granulosa cell marker (FOXL2, nuclear staining). No differences were observed between KO and WT ovaries; in both KO and WT ovaries, primordial follicles were forming at P0 (see higher magnification, boxed).
(TIF)

S5 Fig Genes differentially expressed in *Dmxl2* KO gonads at P0. RT-qPCR validation of microarray results for *Aph1b*, *Fez1*, *Coro2b* and *Fbxw8*. Different letters indicate significant differences between conditions.
(TIF)

S6 Fig Histology of ovaries from six-month-old mice with a cell-specific *Dmxl2* KO. Ovaries from the different genotypes (control, granulosa cell cKO, germ cell cKO and dcKO) were similar in size and displayed normal folliculogenesis. All stages were observed, from primordial follicles to antral follicles. Prl: primordial follicle; Pr: primary follicle; Sec: secondary follicle; PA: pre-antral follicle; A: antral follicle.
(TIF)

S7 Fig Histology of testes and associated sperm parameters in six-month-old mice harboring a cell-specific *Dmxl2* KO. **(A)** Histology of testes from six-month-old mice with a cell-specific *Dmxl2* KO. All spermatogenic stages are visible in all four genotypes. In germ cell cKO and dcKO testes, the lumen of a large proportion of seminiferous tubule is much less visible than that of the control and Sertoli cell *c*KO testes. **(B)** Sperm parameters and testicular weight for mice with a cell-specific *Dmxl2* KO. The epididymal sperm concentration of mice with cell-specific mutations was not significantly different from that of control *Dmxl2*^loxP/loxP^ mice, and no effect on sperm motility was observed. For testis weight, only germ cell cKO testes differed in weight from the control, being slightly lighter. Significant differences are represented by an asterisk.
(TIF)

S8 Fig Histology of testes from seven-week-old mice harboring a cell-specific *Dmxl2* KO. In germ cell cKO and dcKO testes, the lumen diameter of the seminiferous tubule was smaller, whereas the area occupied by Sertoli cell cytoplasm was larger than that in control and Sertoli cell cKO testes.
(TIF)

S9 Fig Cellular expression of the 363 genes differentially expressed in *Dmxl2* dcKO testes. Differential expression analyses identified 363 genes differentially expressed in the testes of seven-week-old *Dmxl2* dcKO and control mice (adjusted pValue<0.05). This list of genes was then compared with the data of Soumillon et al. [31] (see S1 File, “Reported to GSE43717” tab) who reported expression levels (fpkm) for all these genes in purified Sertoli cells, spermatogonia, spermatocytes, spermatids and spermatozoa. A heat map was generated for these 363 genes, based on their level of expression in each cell type. Genes were then sorted into two groups, **(A)** upregulated or **(B)** downregulated in *Dmxl2* dcKO testes.
(TIF)

S10 Fig Sertoli cell detection and counting in *Dmxl2* dcKO testes. **(A)** Immunohistochemistry was used to detect SOX9-positive cells (brown) in control and dcKO testes seven weeks after birth. **(B)** The SOX9-positive cells were counted in each genotype, and the results are expressed per mm^2^ of seminiferous tubules. No significant difference was observed between the two genotypes (pValue = 0.28).
(TIF)
