# Supplementary material for: Dual role of DMXL2 in olfactory information transmission and the first wave of spermatogenesis
Source: PLoS Genet. 2019 Feb 8;15(2):e1007909. doi: 10.1371/journal.pgen.1007909 (PMC6383954; doi:10.1371/journal.pgen.1007909)
Supplement: S1 Table — (DOCX) [file pgen.1007909.s001.docx]

**S1 Table: List of primers.**

| **Gene** | **Forward (5' - 3')** | **Reverse (5' - 3')** |
| --- | --- | --- |
| **Genotyping** |  |  |
| *Amh-CRE* |  |  |
| *Amhr2-CRE* | CGCATTGTCTGAGTAGGTGT | GAAACGCAGCTCGGCCAGC |
| *Dmxl2* (WT) | AGCAGTCTCTCCAGCCTCAG (1) | AAGGTACCTGCAAGGGTAAAA (2) |
| *Dmxl2* (tm1a) | AGCAGTCTCTCCAGCCTCAG (1) | TCGTGGTATCGTTATGCGCC (3) |
| *Vasa-CRE* | CACGTGCAGCCGTTTAAGCCGCGT | TTCCCATTCTAAACAACACCCTGAA |
| **PCR** |  |  |
| *Dmxl2* | TTGCCACTGCTGGAAAGGAT (a) | AGCCCGTCTCCTCTTCACTTC (b) |
| *Gapdh* | AGCCCATCACCATCTTCCAG | TCATGACCACAGTCCATGCCA |
| **qPCR** |  |  |
| *ActB* | TGGGCATGGGTCAGAAGGAT | TGACGATGCCGTGCTCGAT |
| *Aph1b* | CTGCTGTCATCCGTTTTTTGG | CACTCCGAAGATGAGCAGGTAA |
| *Axl* | GGGTATCTGGCTGGGAAAGTC | GGGTGTGAGGAAGGAGCTTTT |
| *Coro2b* | ACAGTCACCGCCCTTTGG | TCTTGCCTGCCCACCCT |
| *Dmxl2 ex7* | ACCCTATGACTGGCTGGAAGTC | TGTGCCAAGTAAACAAAGGAGAAC |
| *Fbxw8* | GAAGGTTCCCTATCTTTCGTTTTG | CAGAAGCCGTGGCAACAAT |
| *Fez1* | CTGGCCCGCAGAGATGAG | GCTCCCTCTGCTTGTTCTGAA |
| *Gas6* | GAATTTGACTTCCGCACTTTTGA | GCCGCCCAGCTCTTAGG |
| *Mertk* | ACTGCCGAGAAGTGGGAAGA | AGAGAATGGCCTGTGGTTGACT |
| *Pros1* | GAAAATTCAGTGGTGGCTCGAT | TCCACAGTTCCAGTCCATTTCTG |
| *Tyro3* | ACATTCTGGGCCACCTGTCT | ACCTCAGGGCTGCCACTCT |
| *Ywhaz* | GGAGCTGAGCTGTCGAATGA | GACCCTCCACGATGACCTA |
|  |  |  |
|  |  |  |
|  |  |  |
|  |  |  |
