# Supplementary material for: Dual role of DMXL2 in olfactory information transmission and the first wave of spermatogenesis
Source: PLoS Genet. 2019 Feb 8;15(2):e1007909. doi: 10.1371/journal.pgen.1007909 (PMC6383954; doi:10.1371/journal.pgen.1007909)
Supplement: S2 Table — We found that 51 genes were differentially regulated between KO and WT ovaries (ordered according to their fold-change in expression): 28 were downregulated in KO ovaries, whereas 23 were upregulated. a Two of the upregulated genes were represented by several probes (B3gat1 and Tpm1). (DOCX) [file pgen.1007909.s002.docx]

**S2 Table:** **List of deregulated genes in *Dmxl2* KO ovaries at birth (Adjusted pValue <0.1).**

| **Gene name**^a^ | **Mus musculus gene ID** | **Probe name** | **Fold change** | **Adj. pValue** |
| --- | --- | --- | --- | --- |
| **Down-regulated in KO ovaries compared to WT** | | | | |
| *Angptl4* | ENSMUSG00000002289 | ILMN_2759365 | 0.302 | 0.036 |
| *Akr1c12/13* | ENSMUSG00000021211 | ILMN_1254015 | 0.506 | 0.046 |
| *Leng9* | ENSMUSG00000043432 | ILMN_2757682 | 0.524 | 0.042 |
| *Trpc7* | ENSMUSG00000021541 | ILMN_1242557 | 0.537 | 0.002 |
| *Sqle* | ENSMUSG00000022351 | ILMN_2600348 | 0.564 | 0.042 |
| *Tmed1* | ENSMUSG00000032180 | ILMN_1214974 | 0.610 | 0.071 |
| *Ntn3* | ENSMUSG00000079662 | ILMN_2615312 | 0.658 | 0.029 |
| *Cftr* | ENSMUSG00000041301 | ILMN_1259577 | 0.659 | 0.029 |
| *Prmt6* | ENSMUSG00000049300 | ILMN_3007271 | 0.678 | 0.057 |
| *Nfe2l2* | ENSMUSG00000015839 | ILMN_1224128 | 0.686 | 0.099 |
| *Fam117a* | ENSMUSG00000038893 | ILMN_1224162 | 0.690 | 0.094 |
| *Vat1l* | ENSMUSG00000046844 | ILMN_1226356 | 0.709 | 0.042 |
| *Fbxw8* | ENSMUSG00000032867 | ILMN_1251236 | 0.710 | 0.029 |
| *Fadd* | ENSMUSG00000031077 | ILMN_1223126 | 0.724 | 0.071 |
| *Chaf1b* | ENSMUSG00000022945 | ILMN_2664686 | 0.733 | 0.071 |
| *0610007P14Rik* | ENSMUSG00000021252 | ILMN_2771349 | 0.735 | 0.043 |
| *Slc25a5* | ENSMUSG00000016319 | ILMN_2660099 | 0.738 | 0.071 |
| *Dmxl2* | ENSMUSG00000041268 | ILMN_2467185 | 0.740 | 0.036 |
| *Aqp5* | ENSMUSG00000044217 | ILMN_3115472 | 0.746 | 0.044 |
| *Pramef8* | ENSMUSG00000046862 | ILMN_1212648 | 0.750 | 0.099 |
| *Hnrnph1* | ENSMUSG00000007850 | ILMN_2688923 | 0.752 | 0.051 |
| *E2f6* | ENSMUSG00000057469 | ILMN_2723931 | 0.753 | 0.056 |
| *Mtch1* | ENSMUSG00000024012 | ILMN_1239041 | 0.757 | 0.099 |
| *Srm* | ENSMUSG00000006442 | ILMN_1225880 | 0.763 | 0.073 |
| *Rbbp9* | ENSMUSG00000027428 | ILMN_1222004 | 0.767 | 0.029 |
| *Syt16* | ENSMUSG00000044912 | ILMN_1246983 | 0.780 | 0.036 |
| *Timeless* | ENSMUSG00000039994 | ILMN_3097131 | 0.786 | 0.099 |
| *Mus81* | ENSMUSG00000024906 | ILMN_2651462 | 0.836 | 0.099 |
| **Up-regulated in KO ovaries compared to WT** | | | | |
| *Vwa8* | ENSMUSG00000058997 | ILMN_1226962 | 1.224 | 0.057 |
| *Zfp612* | ENSMUSG00000044676 | ILMN_2491719 | 1.226 | 0.094 |
| *B3gat1* | ENSMUSG00000045994 | ILMN_2853122 | 1.333 | 0.034 |
| *Fez1* | ENSMUSG00000032118 | ILMN_1213056 | 1.338 | 0.099 |
| *Tfdp2* | ENSMUSG00000032411 | ILMN_2488405 | 1.381 | 0.043 |
| *Dcp1a* | ENSMUSG00000021962 | ILMN_2783429 | 1.390 | 0.071 |
| *Tsc1* | ENSMUSG00000026812 | ILMN_2465589 | 1.403 | 0.090 |
| *Hdc* | ENSMUSG00000027360 | ILMN_1256122 | 1.426 | 0.043 |
| *Elf4* | ENSMUSG00000031103 | ILMN_1232925 | 1.431 | 0.088 |
| *Coro2b* | ENSMUSG00000041729 | ILMN_2902228 | 1.465 | 0.036 |
| *Morn5* | ENSMUSG00000026894 | ILMN_1253329 | 1.466 | 0.036 |
| *Vat1* | ENSMUSG00000034993 | ILMN_2440530 | 1.471 | 0.045 |
| *B3gat1* | ENSMUSG00000045994 | ILMN_2708717 | 1.498 | 0.036 |
| *Hdac4* | ENSMUSG00000026313 | ILMN_1219682 | 1.503 | 0.010 |
| *Tpm1* | ENSMUSG00000032366 | ILMN_3007072 | 1.504 | 0.036 |
| *Smc1b* | ENSMUSG00000022432 | ILMN_2603908 | 1.662 | 0.088 |
| *Spg21* | ENSMUSG00000032388 | ILMN_2705673 | 1.677 | 0.082 |
| *Aph1b* | ENSMUSG00000032375 | ILMN_2588682 | 2.100 | 0.036 |
| *Hbb-bs/bt* | ENSMUSG00000052305 | ILMN_1239117 | 2.147 | 0.043 |
| *Srpr* | ENSMUSG00000032042 | ILMN_2747480 | 2.166 | 0.036 |
| *Adpgk* | ENSMUSG00000025236 | ILMN_2633148 | 2.171 | 0.099 |
| *Tpm1* | ENSMUSG00000032366 | ILMN_2518346 | 2.193 | 0.088 |
| *Pdcd7* | ENSMUSG00000041837 | ILMN_1240075 | 2.404 | 0.099 |
| *Kif23* | ENSMUSG00000032254 | ILMN_1220121 | 2.707 | 0.099 |
| *C920006O11Rik* | ENSMUSG00000097574 | ILMN_1213875 | 3.612 | 0.042 |
