# Supplementary material for: Dual role of DMXL2 in olfactory information transmission and the first wave of spermatogenesis
Source: PLoS Genet. 2019 Feb 8;15(2):e1007909. doi: 10.1371/journal.pgen.1007909 (PMC6383954; doi:10.1371/journal.pgen.1007909)
Supplement: S3 Table — We found that 12 genes were differentially regulated between KO and WT testes (ordered according to their fold-change in expression). Three were downregulated and the other nine were upregulated in KO testes. (DOCX) [file pgen.1007909.s003.docx]

**S3 Table:** **List of deregulated genes in *Dmxl2* KO testes at birth (Adjusted pValue < 0.1).**

| **Gene name** | **Mus musculus gene ID** | **Probe name** | **Fold change** | **Adj. pValue** |
| --- | --- | --- | --- | --- |
| **Down-regulated in KO testes compared to WT** | | | | |
| *Car12* | ENSMUSG00000032373 | ILMN_2891583 | 0.722 | 0.079 |
| *Cdc6* | ENSMUSG00000017499 | ILMN_2597255 | 0.737 | 0.062 |
| *2210015D19Rik* | ENSMUSG00000083844 | ILMN_1259535 | 0.789 | 0.058 |
| **Up-regulated in KO testes compared to WT** | | | | |
| *Tspan15* | ENSMUSG00000037031 | ILMN_2422360 | 1.403 | 0.042 |
| *Bmf* | ENSMUSG00000040093 | ILMN_2497190 | 1.405 | 0.058 |
| *Tipin* | ENSMUSG00000032397 | ILMN_1234909 | 1.452 | 0.084 |
| *Fez1* | ENSMUSG00000032118 | ILMN_1213056 | 1.539 | 0.042 |
| *Nnmt* | ENSMUSG00000032271 | ILMN_2544305 | 1.624 | 0.042 |
| *Srpr* | ENSMUSG00000032042 | ILMN_2747480 | 2.109 | 0.058 |
| *Aph1b* | ENSMUSG00000032375 | ILMN_2588682 | 2.218 | 0.042 |
| *Pif1* | ENSMUSG00000041064 | ILMN_2714678 | 2.947 | 0.042 |
| *Kif23* | ENSMUSG00000032254 | ILMN_1220121 | 4.496 | 0.042 |
