# Supplementary material for: Dual role of DMXL2 in olfactory information transmission and the first wave of spermatogenesis
Source: PLoS Genet. 2019 Feb 8;15(2):e1007909. doi: 10.1371/journal.pgen.1007909 (PMC6383954; doi:10.1371/journal.pgen.1007909)
Supplement: S4 Table — (DOCX) [file pgen.1007909.s004.docx]

**S4 Table: Weight of the testes and body weight in mice of the different genotypes at seven weeks after birth.**

| Animal | Genotype | Testis weight (g) | Body weight (g) | Testis weight / body weight ratio (%) |
| --- | --- | --- | --- | --- |
| # 1 | Dmxl2 ^loxP/loxP^ | 0,2470 | 23,74 | 1,040% |
| # 2 | Dmxl2 ^loxP/loxP^ | 0,2420 | 24,39 | 0,992% |
| # 3 | Dmxl2 ^loxP/loxP^ | 0,1930 | 23,07 | 0,837% |
| # 4 | Dmxl2 ^loxP/loxP^ | 0,2400 | 27,42 | 0,875% |
| # 5 | Dmxl2 ^loxP/loxP^ | 0,2580 | 27,04 | 0,954% |
| # 6 | Dmxl2 ^loxP/loxP^ | 0,2360 | 25,47 | 0,927% |
| # 7 | Dmxl2 ^loxP/loxP^ | 0,2530 | 22,88 | 1,106% |
| # 8 | Dmxl2 ^loxP/loxP^ | 0,1700 | 22,43 | 0,758% |
| # 9 | Dmxl2 ^loxP/loxP^ | 0,2220 | 25,54 | 0,869% |
| # 10 | Dmxl2 ^loxP/loxP^ | 0,2260 | 23,21 | 0,974% |
| # 11 | Dmxl2 ^loxP/loxP^ | 0,1610 | 22,25 | 0,724% |
| # 12 | Dmxl2 ^loxP/loxP^ | 0,1590 | 23,61 | 0,673% |
| # 13 | Dmxl2 ^loxP/loxP^ | 0,1830 | 22,68 | 0,807% |
| # 14 | Dmxl2 ^loxP/loxP^ | 0,2040 | 22,72 | 0,898% |
| # 15 | Dmxl2 ^loxP/loxP^ ; Amh-Cre | 0,1770 | 27,32 | 0,648% |
| # 16 | Dmxl2 ^loxP/loxP^ ; Amh-Cre | 0,1730 | 22,97 | 0,753% |
| # 17 | Dmxl2 ^loxP/loxP^ ; Amh-Cre | 0,2040 | 23,47 | 0,869% |
| # 18 | Dmxl2 ^loxP/loxP^ ; Amh-Cre | 0,2290 | 23,65 | 0,968% |
| # 19 | Dmxl2 ^loxP/loxP^ ; Amh-Cre | 0,2030 | 21,47 | 0,946% |
| # 20 | Dmxl2 ^loxP/loxP^ ; Amh-Cre | 0,2040 | 21,31 | 0,957% |
| # 21 | Dmxl2 ^loxP/loxP^ ; Amh-Cre | 0,1970 | 20,13 | 0,979% |
| # 22 | Dmxl2 ^loxP/loxP^ ; Amh-Cre | 0,1930 | 26,35 | 0,732% |
| # 23 | Dmxl2 ^loxP/loxP^ ; Amh-Cre | 0,1990 | 24,17 | 0,823% |
| # 24 | Dmxl2 ^loxP/loxP^ ; Amh-Cre | 0,2350 | 23,84 | 0,986% |
| # 25 | Dmxl2 ^loxP/loxP^ ; Amh-Cre | 0,1760 | 23,83 | 0,739% |
| # 26 | Dmxl2 ^loxP/loxP^ ; Amh-Cre | 0,1930 | 23,99 | 0,805% |
| # 27 | Dmxl2 ^loxP/loxP^ ; Amh-Cre | 0,1970 | 23,66 | 0,833% |
| # 28 | Dmxl2 ^loxP/-^ ; Vasa-Cre | 0,2420 | 24,26 | 0,998% |
| # 29 | Dmxl2 ^loxP/-^ ; Vasa-Cre | 0,2260 | 24,62 | 0,918% |
| # 30 | Dmxl2 ^loxP/-^ ; Vasa-Cre | 0,2250 | 22,67 | 0,993% |
| # 31 | Dmxl2 ^loxP/-^ ; Vasa-Cre | 0,1780 | 24,2 | 0,736% |
| # 32 | Dmxl2 ^loxP/-^ ; Vasa-Cre ; Amh-Cre | 0,2160 | 22,13 | 0,976% |
| # 33 | Dmxl2 ^loxP/-^ ; Vasa-Cre ; Amh-Cre | 0,1630 | 19,96 | 0,817% |
| # 34 | Dmxl2 ^loxP/-^ ; Vasa-Cre ; Amh-Cre | 0,2270 | 23,77 | 0,955% |
| # 35 | Dmxl2 ^loxP/-^ ; Vasa-Cre ; Amh-Cre | 0,2050 | 23,64 | 0,867% |
