# Supplementary material for: Dual role of DMXL2 in olfactory information transmission and the first wave of spermatogenesis
Source: PLoS Genet. 2019 Feb 8;15(2):e1007909. doi: 10.1371/journal.pgen.1007909 (PMC6383954; doi:10.1371/journal.pgen.1007909)
Supplement: S5 Table — (DOCX) [file pgen.1007909.s005.docx]

**S5 Table: List of antibodies and their condition of use.**

| **Primary antibody** | **Reference** | **Source** | **Dilution** |
| --- | --- | --- | --- |
| *Immunohistology* |  |  |  |
| C-FOS | Ab-5 (Calbiochem) | Rabbit | 1:2 000 |
| DMXL2 | HPA039375 (Sigma) | Rabbit | 1:200 |
| FOXL2 | Boulanger *et al*., 2014 | Rabbit | 1:100 |
| VASA | ab13840 (Abcam) | Rabbit | 1:500 |
| LAMININ | L9393 (Sigma-Aldrich) | Rabbit | 1:25 |
| Cleaved-CASPASE 3 | 9661 (Cell signaling) | Rabbit | 1:200 |
| SOX9 | Notarnicola et al., 2006 [65] | Rabbit | 1 :500 |
|  |  |  |  |
| *Western Blot* |  |  |  |
| DMXL2 | HPA039375 (Sigma) | Rabbit | 1:500 |
| GAPDH | sc-25778 (SantaCruz) | Rabbit | 1:1 000 |
|  |  |  |  |
| **Secondary antibody** | **Reference** | **Conjugate** | **Dilution** |
| *Immunohistology* |  |  |  |
| Anti-rabbit IgG 594 | 072-09-15-06 (KPL) | Dye 594 | 1 : 200 |
| Anti-rabbit IgG-Biotinylated | BA-1100 (Vector laboratory) | Biotin | 1 :200 |
|  |  |  |  |
| *Western Blot* |  |  |  |
| Goat anti-rabbit IgG-HRP | sc-2054 (SantaCruz) | Peroxidase | 1:5 000 |
| Monkey anti-rabbit IgG-HRP | UP-559721 (Interchim) | Peroxidase | 1:10 000 |
| Goat anti-mouse IgG-HRP | sc-2055 (SantaCruz) | Peroxidase | 1:5 000 |
|  |  |  |  |
|  |  |  |  |
|  |  |  |  |
|  |  |  |  |
