# Supplementary figures and images for: Dual role of DMXL2 in olfactory information transmission and the first wave of spermatogenesis
Source: PLoS Genet. 2019 Feb 8;15(2):e1007909. doi: 10.1371/journal.pgen.1007909 (PMC6383954; doi:10.1371/journal.pgen.1007909)

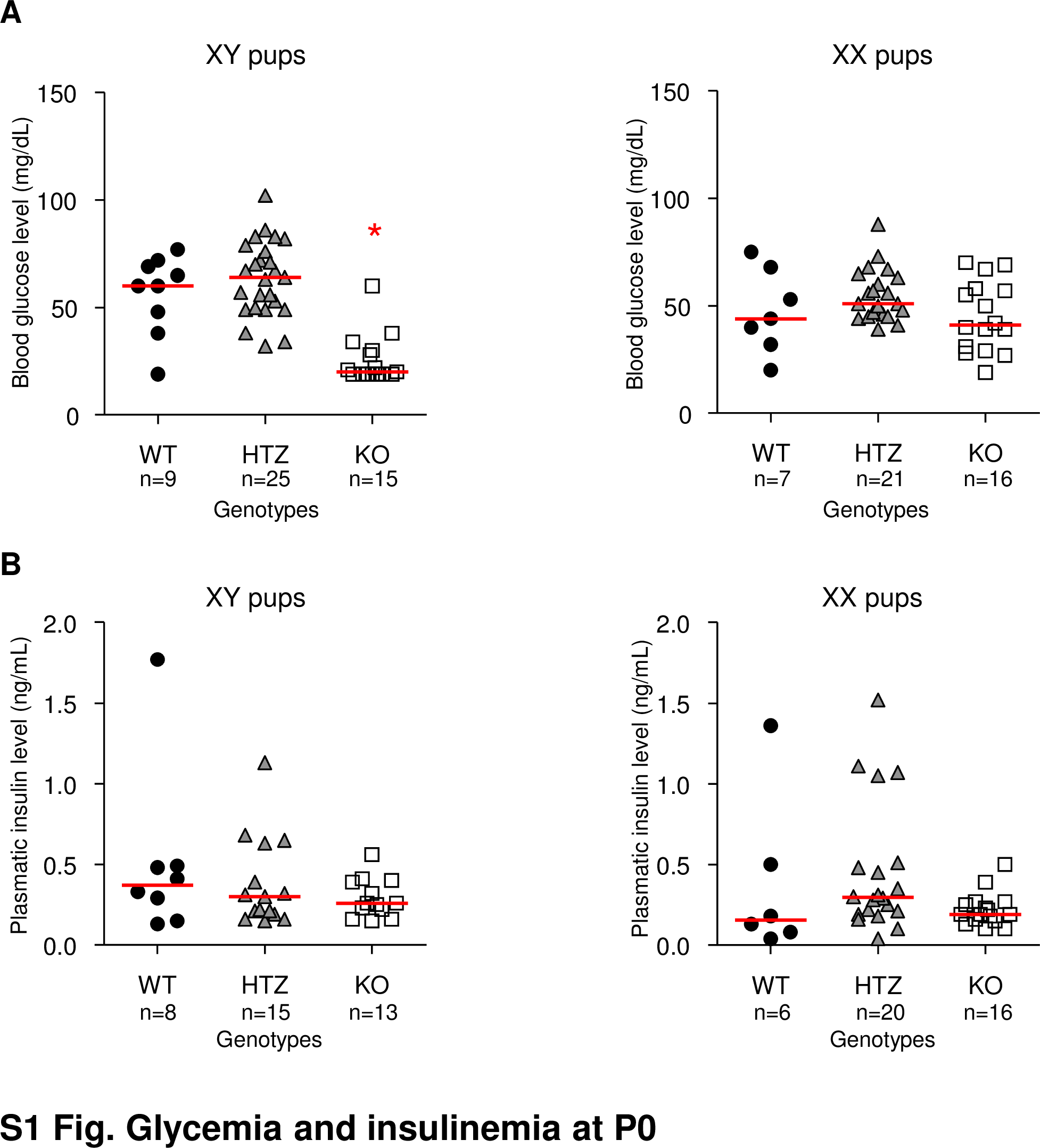

Supplement: S1 Fig — (A) Blood glucose concentration at P0. Only Dmxl2 KO XY pups (males) are hypoglycemic (20 mg/dl), with significantly lower (66.7% lower) blood glucose concentrations than WT pups (60 mg/dL). (B) Plasma insulin concentration at P0. Insulinemia was similar in Dmxl2 WT, HTZ and KO pups (0.25 ng/ml). Significant differences are indicated by asterisks. (TIF) [file pgen.1007909.s012.tif]

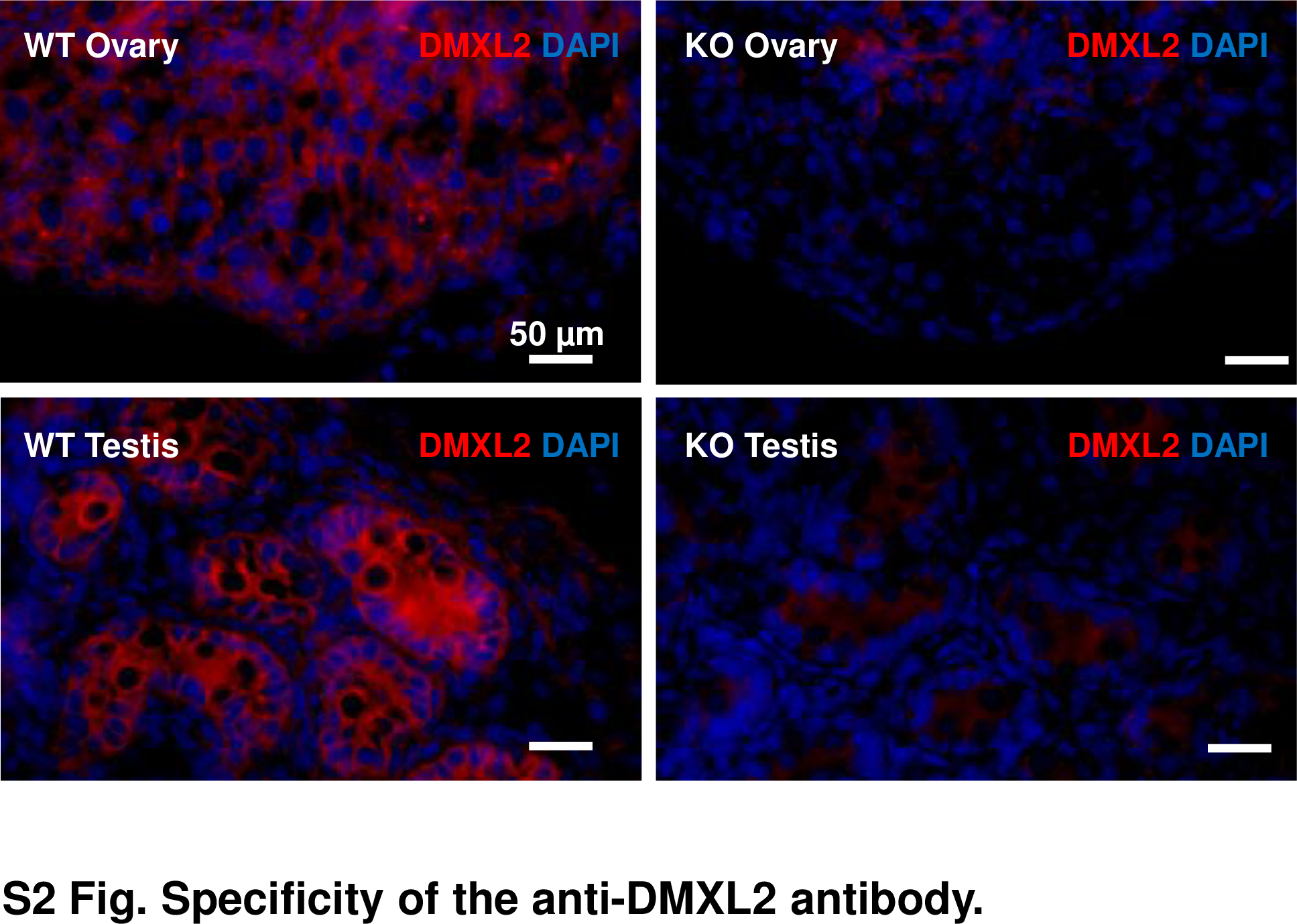

Supplement: S2 Fig — At P0, DMXL2 was detected in the cytoplasm of germ cells and somatic cells in WT ovaries and testes. No staining was observed in Dmxl2 KO gonads other than a faint background in male germ cells. (TIF) [file pgen.1007909.s013.tif]

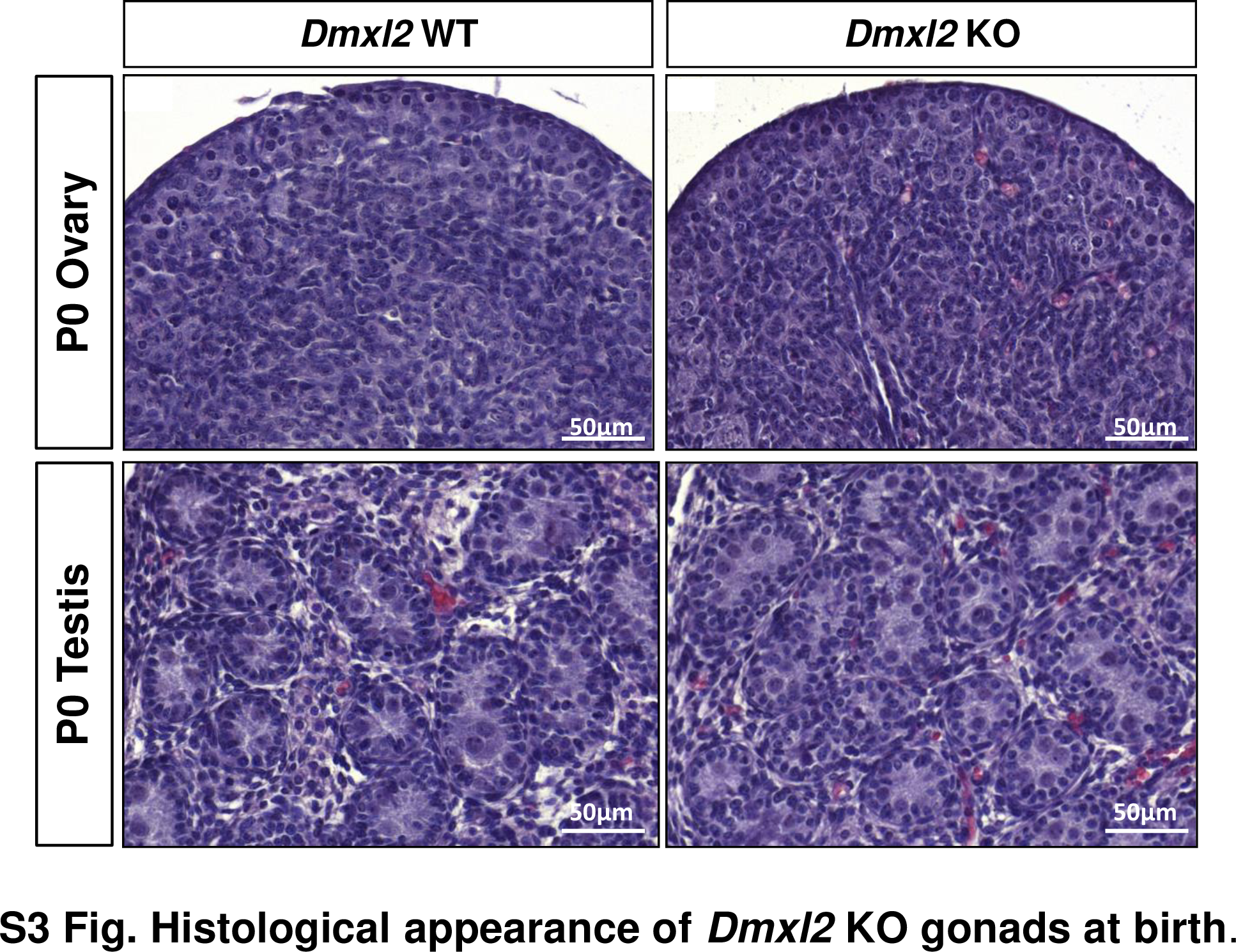

Supplement: S3 Fig — Hematoxylin and eosin staining revealed no obvious differences between Dmxl2 KO and control gonads at birth, in terms of size and organization. The ovaries had germ cell nests in the cortex, and seminiferous cords were evident in the testes. (TIF) [file pgen.1007909.s014.tif]

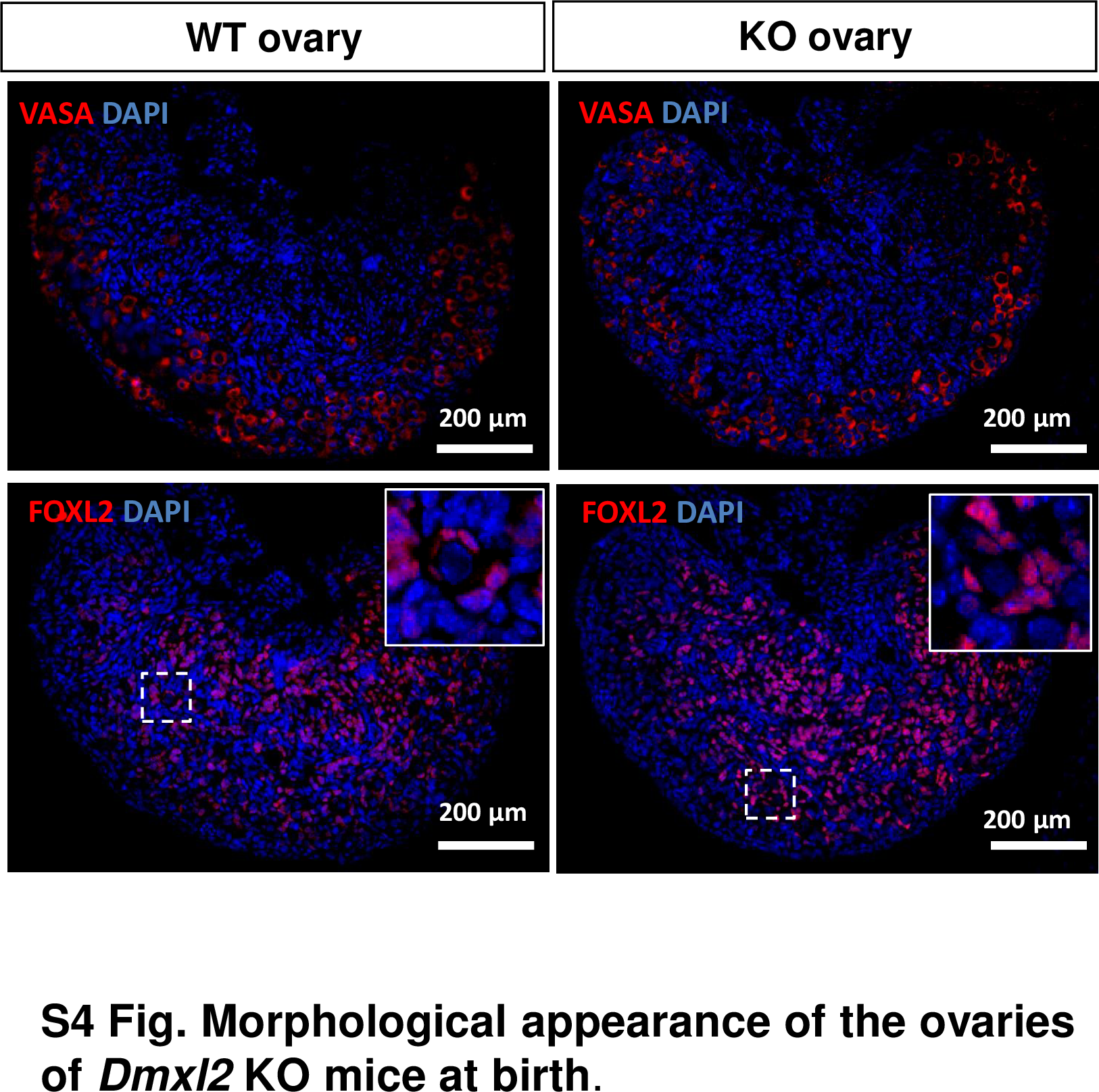

Supplement: S4 Fig — Immunofluorescence studies were performed with a germ cell marker (VASA, cytoplasmic staining) and a pre-granulosa cell marker (FOXL2, nuclear staining). No differences were observed between KO and WT ovaries; in both KO and WT ovaries, primordial follicles were forming at P0 (see higher magnification, boxed). (TIF) [file pgen.1007909.s015.tif]

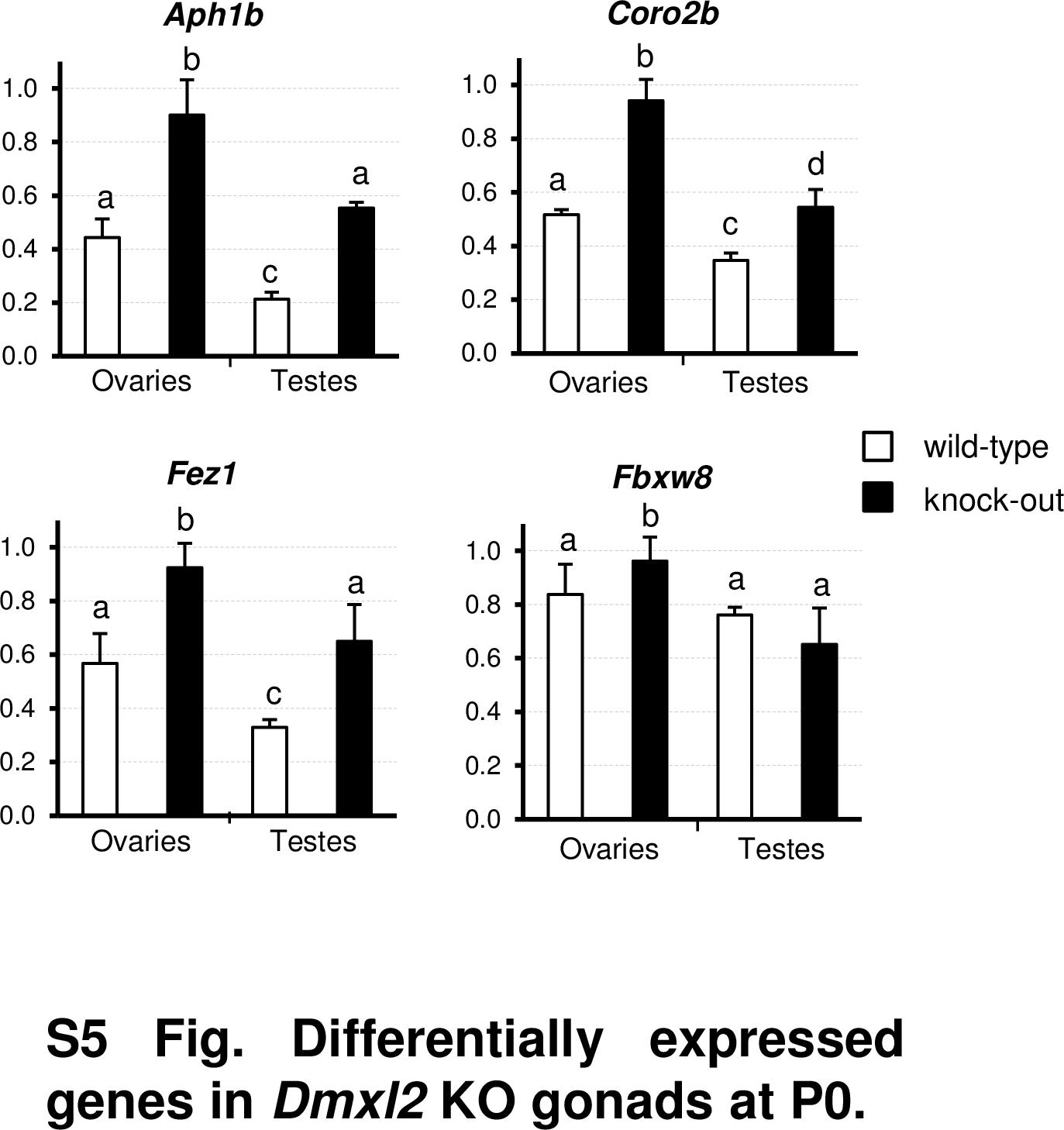

Supplement: S5 Fig — RT-qPCR validation of microarray results for Aph1b, Fez1, Coro2b and Fbxw8. Different letters indicate significant differences between conditions. (TIF) [file pgen.1007909.s016.tif]

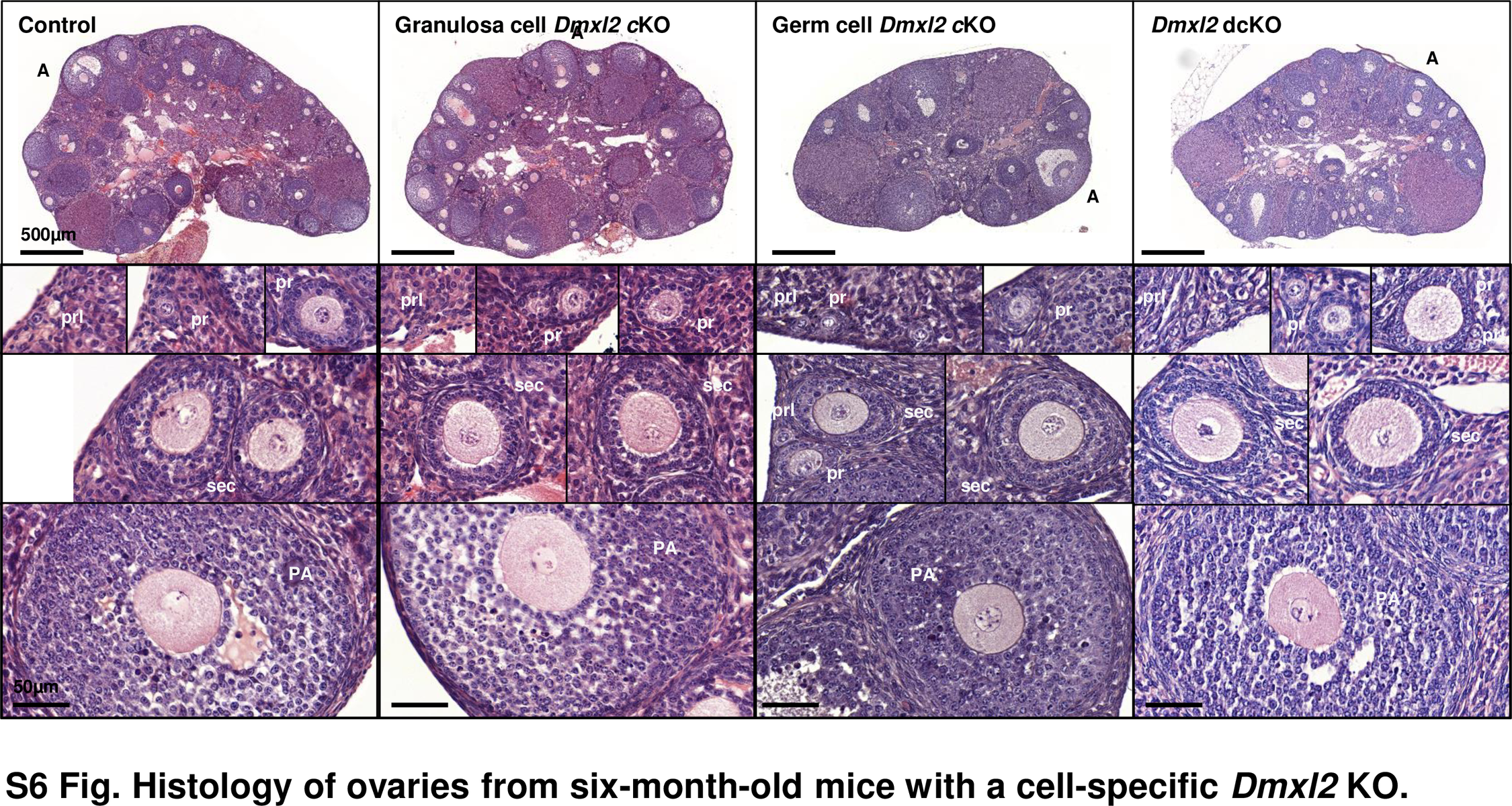

Supplement: S6 Fig — Ovaries from the different genotypes (control, granulosa cell cKO, germ cell cKO and dcKO) were similar in size and displayed normal folliculogenesis. All stages were observed, from primordial follicles to antral follicles. Prl: primordial follicle; Pr: primary follicle; Sec: secondary follicle; PA: pre-antral follicle; A: antral follicle. (TIF) [file pgen.1007909.s017.tif]

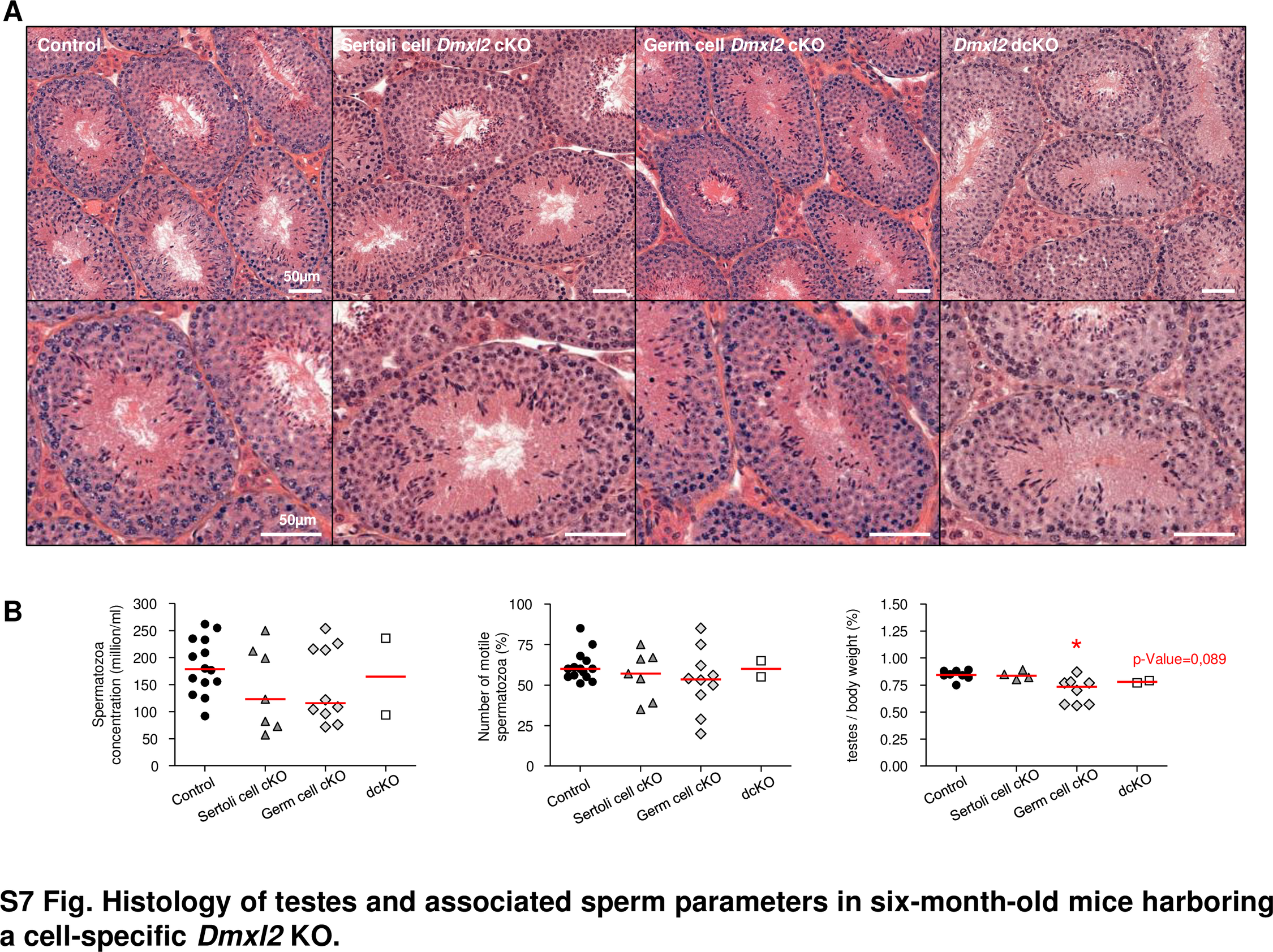

Supplement: S7 Fig — (A) Histology of testes from six-month-old mice with a cell-specific Dmxl2 KO. All spermatogenic stages are visible in all four genotypes. In germ cell cKO and dcKO testes, the lumen of a large proportion of seminiferous tubule is much less visible than that of the control and Sertoli cell cKO testes. (B) Sperm parameters and testicular weight for mice with a cell-specific Dmxl2 KO. The epididymal sperm concentration of mice with cell-specific mutations was not significantly different from that of control Dmxl2loxP/loxP mice, and no effect on sperm motility was observed. For testis weight, only germ cell cKO testes differed in weight from the control, being slightly lighter. Significant differences are represented by an asterisk. (TIF) [file pgen.1007909.s018.tif]

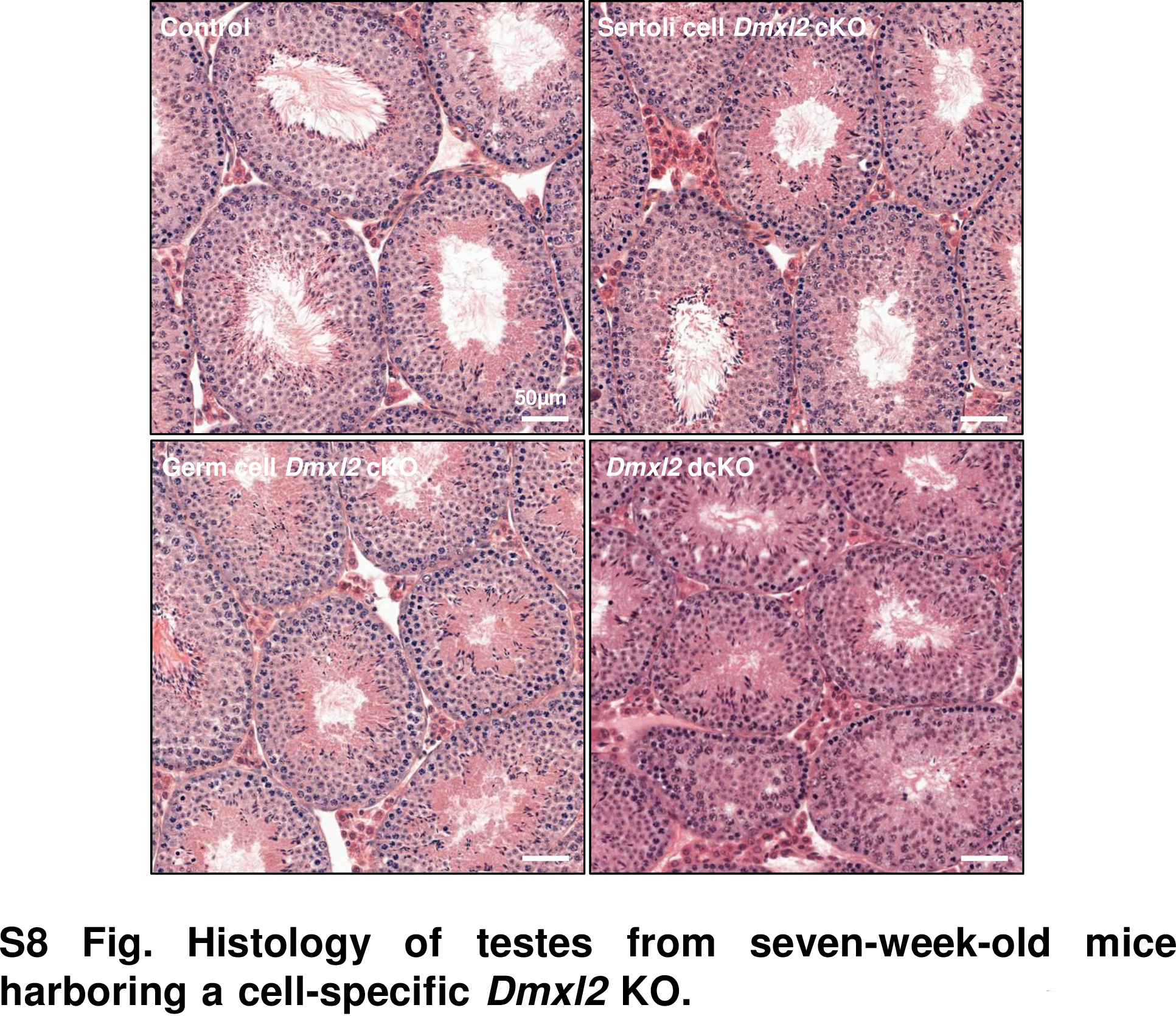

Supplement: S8 Fig — In germ cell cKO and dcKO testes, the lumen diameter of the seminiferous tubule was smaller, whereas the area occupied by Sertoli cell cytoplasm was larger than that in control and Sertoli cell cKO testes. (TIF) [file pgen.1007909.s019.tif]

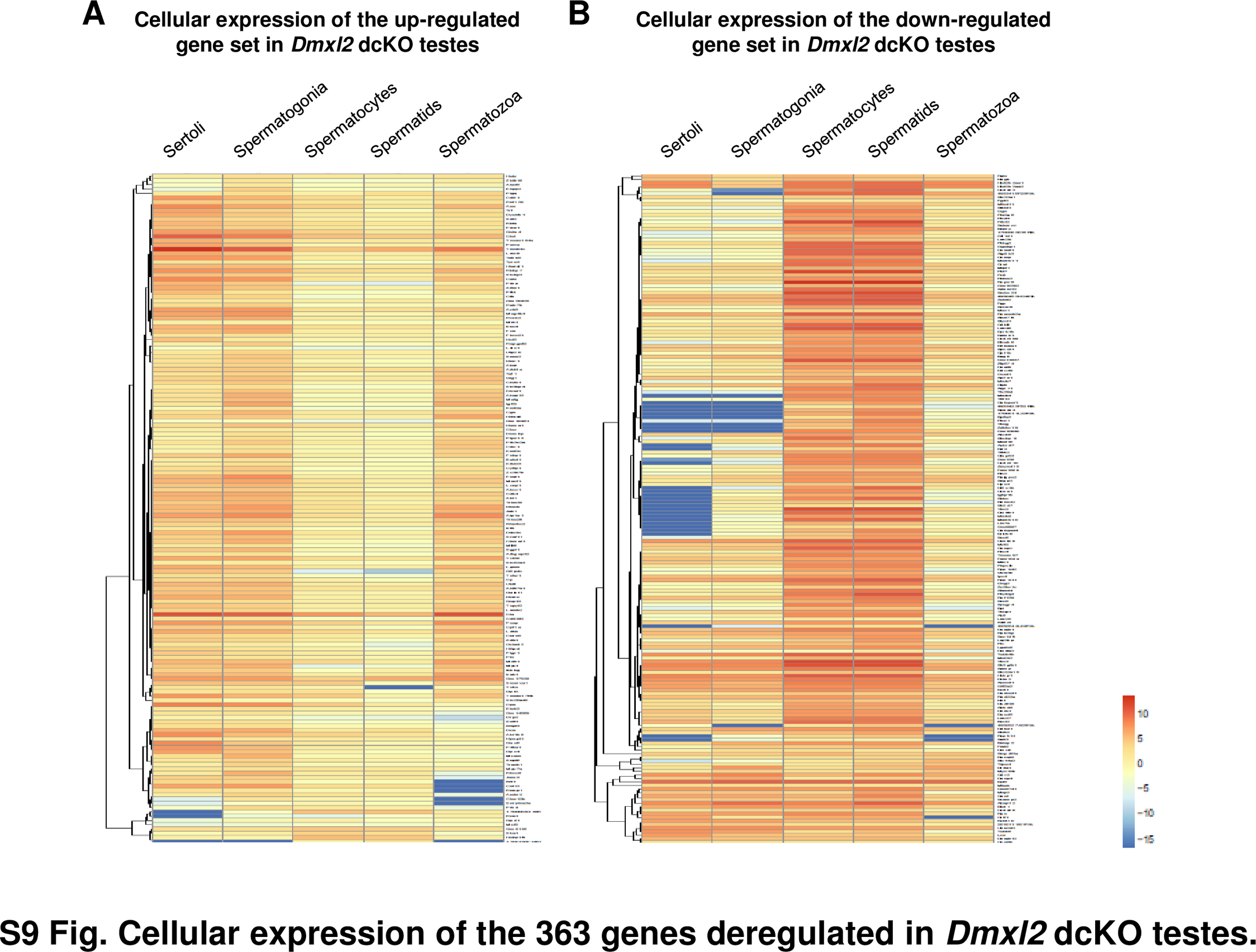

Supplement: S9 Fig — Differential expression analyses identified 363 genes differentially expressed in the testes of seven-week-old Dmxl2 dcKO and control mice (adjusted pValue<0.05). This list of genes was then compared with the data of Soumillon et al. [31] (see S1 File, “Reported to GSE43717” tab) who reported expression levels (fpkm) for all these genes in purified Sertoli cells, spermatogonia, spermatocytes, spermatids and spermatozoa. A heat map was generated for these 363 genes, based on their level of expression in each cell type. Genes were then sorted into two groups, (A) upregulated or (B) downregulated in Dmxl2 dcKO testes. (TIF) [file pgen.1007909.s020.tif]

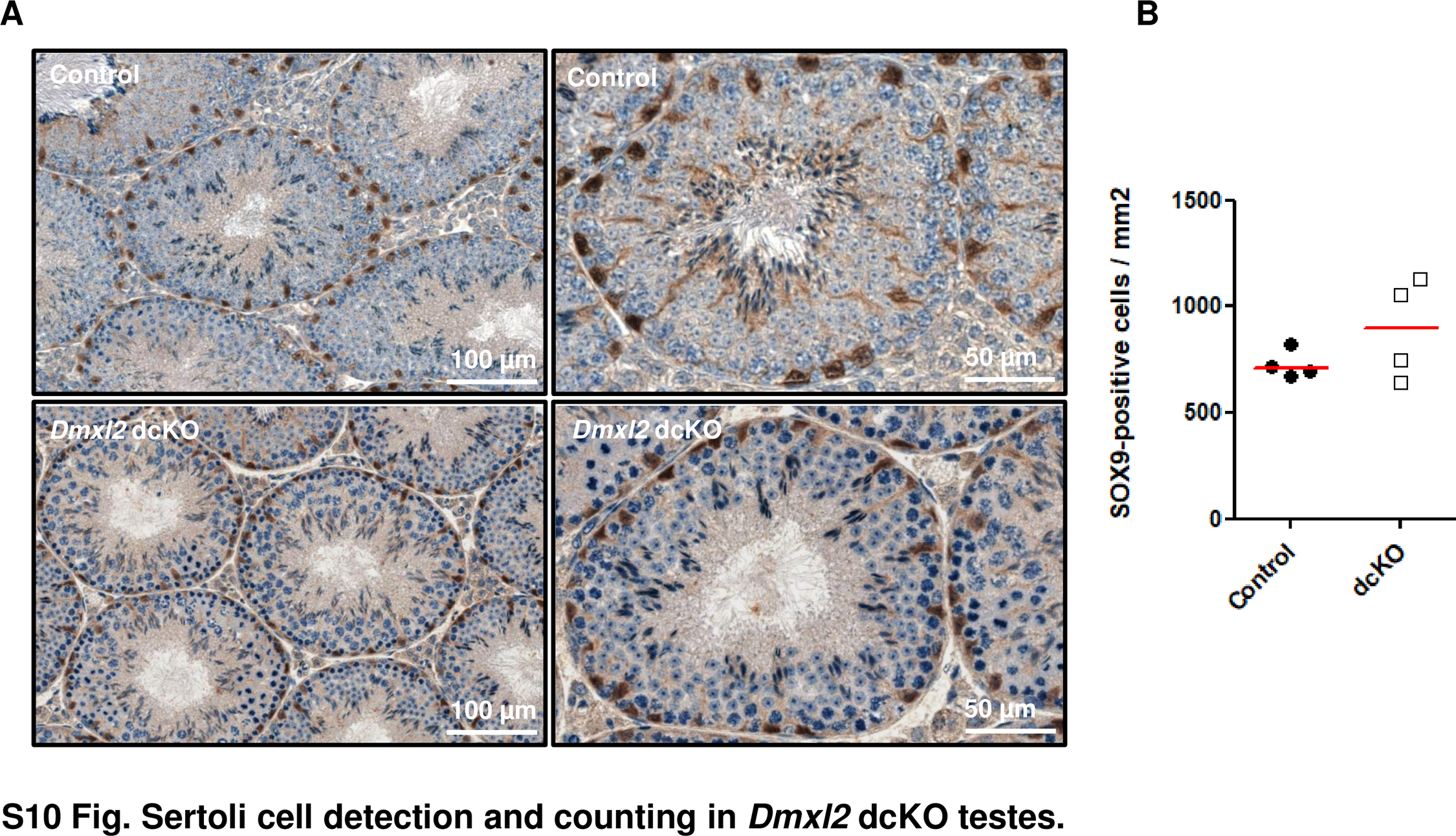

Supplement: S10 Fig — (A) Immunohistochemistry was used to detect SOX9-positive cells (brown) in control and dcKO testes seven weeks after birth. (B) The SOX9-positive cells were counted in each genotype, and the results are expressed per mm2 of seminiferous tubules. No significant difference was observed between the two genotypes (pValue = 0.28). (TIF) [file pgen.1007909.s021.tif]
